# The Effect of Carbon Nanofibers on the Microstructure, Chemistry, and Pore Structure of Concrete Made with Fine Recycled Concrete Aggregates

**DOI:** 10.3390/nano15040253

**Published:** 2025-02-07

**Authors:** Nathanial Buettner, Gass Iyacu, Giovanni Dal Poggetto, Ange-Therese Akono

**Affiliations:** 1 Department of Civil and Environmental Engineering, Northwestern University, Evanston, IL 60208, USA; nathanial.buettner@northwestern.edu (N.B.); gass.iyacu@northwestern.edu (G.I.); 2 Department of Civil, Construction, and Environmental Engineering, North Carolina State University, Raleigh, NC 27695, USA; gdalpog@ncsu.edu

**Keywords:** sustainable concrete, nanomaterial modification, durability enhancement, pore structure optimization

## Abstract

Recycled aggregate concrete (RAC) is produced using recycled concrete aggregates (RCAs) obtained from crushed old concrete. Although RCAs offer a sustainable alternative to natural aggregates, the poor durability and mechanical performance of RAC limit its widespread application. This study investigated the enhancement of RAC’s durability and performance through the incorporation of carbon nanofibers (CNFs). A novel processing method was developed to prepare high-slump CNF-modified RAC, and its chemistry, pore structure, and microstructure were analyzed using backscattered scanning electron microscopy (SEM), energy-dispersive spectroscopy (EDS), X-ray powder diffraction (XRD), and mercury intrusion porosimetry (MIP). The results showed that CNFs significantly reduced the porosity and permeability, with a decrease in the porosity by 9.0 wt.% and a decrease in the water permeability by 39.3% at an optimal CNF dosage of 0.5% by weight. Furthermore, CNFs promoted the formation of calcium hydroxide and enhanced the densification of the calcium silicate hydrate (C-S-H) matrix, leading to improved resistance against environmental stressors. These findings provide a critical pathway for designing sustainable, high-durability RAC for structural applications.

## 1. Introduction

Concrete is the backbone of modern infrastructure, underpinning the rapid development of cities and urban areas. However, the global reliance on concrete has led to significant environmental challenges, including the depletion of natural resources and the accumulation of construction and demolition waste. Recycling concrete as aggregates for new concrete production presents substantial opportunities to address these sustainability challenges. The use of recycled concrete aggregates (RCAs) reduces both the embodied energy and carbon dioxide footprint of concrete production [1]. Furthermore, incorporating fine recycled concrete aggregates (FRCAs) specifically can mitigate the increasing demand for sand, projected to rise by 45% between 2020 and 2060 [2]. Recycling concrete also alleviates space shortages by reducing the volume of construction and demolition waste sent to landfills [3,4,5,6]. Despite its ecological benefits, recycled aggregate concrete (RAC) faces significant technical challenges. RCAs possess inherent drawbacks such as a higher porosity, lower density, and the presence of residual mortar on their surfaces. These characteristics compromise the interfacial transition zone (ITZ), a critical determinant of concrete strength, resulting in reduced mechanical performance, increased permeability, and diminished durability. Consequently, the brittleness, shrinkage, and cracking tendencies of RAC limit its application in high-performance structural projects [4,5,6].

In recent years, the incorporation of advanced materials has been explored as a means to overcome these limitations. Among these, nanotechnology, particularly the use of carbon nanomaterials (CNMs), has shown remarkable potential. CNMs such as carbon nanotubes, graphene derivatives, and carbon nanofibers are recognized for their exceptional mechanical, thermal, and chemical properties, which can significantly enhance the microstructure and durability of cementitious composites. CNFs, in particular, have gained attention in the context of RAC due to their ability to address the specific weaknesses of RCAs [5,6,7,8,9,10]. CNFs are cylindrical, layered carbon structures with diameters in the orders of a few hundred nanometers, aspect ratios exceeding 100, and exceptional mechanical and physicochemical properties [9]. CNFs can increase the stiffness [11] and fracture resistance [12] of cementitious materials. Additionally, they have been shown to improve the durability of these materials [13,14,15]. In this study, carbon nanofibers (CNFs) were chosen over other nanomaterials for recycled aggregate concrete (RAC) due to their unique combination of properties and compatibility with cementitious systems. CNFs significantly enhanced the mechanical performance, refined the pore structures, and improved the durability of RAC by acting as nanofillers that bridged hydration products and promoted the formation of dense calcium silicate hydrate (C-S-H) gels. Unlike other nanomaterials such as nanosilica or graphene, CNFs offer a superior balance of high tensile strength, stiffness, and chemical stability in the alkaline cement matrix [5,6,7,8,9,10]. Furthermore, CNFs are less prone to dispersion challenges compared to graphene or carbon nanotubes, and their cost-to-performance ratio makes them a more practical option for large-scale applications. Their ability to reduce the porosity and permeability, particularly in the weak interfacial transition zones of RAC, addresses the primary limitations of recycled aggregates more effectively than many other nanomaterials [5,6,9,12].

Despite these advancements, limited research explores using CNFs to enhance the behavior of recycled aggregate concrete. An important reason is that challenges persist in integrating CNFs into RAC. Achieving the uniform dispersion of CNFs in the cementitious matrix is critical for their reinforcing potential. Poor dispersion can lead to agglomeration, reducing the effectiveness of CNFs and introducing performance inconsistencies. Advanced mixing techniques, such as ultrasonic processing and the use of surfactants, are essential to ensure even distribution [5,6,7,8]. Yet, the integration of CNFs into RAC represents a promising avenue for developing sustainable and high-performance concrete.

This study introduced a novel approach to enhance the durability of RAC using CNFs. Building on prior research that demonstrated that the colloidal nanosilica modification of FRCA mortar reduced the porosity and increased the density of C-S-H gels [16], this study hypothesized that CNFs significantly influence the chemistry and multiscale pore structure of RAC. A novel process was developed to prepare CNF-modified FRCA mortars, focusing on characterizing their properties using a multidisciplinary approach. Advanced techniques, including X-ray diffraction analysis (XRD), scanning electron microscopy equipped with energy-dispersive spectroscopy (SEM/EDS), and mercury intrusion porosimetry (MIP), were employed to evaluate the effects of CNFs on the C-S-H chemistry, microstructure, and pore structure of FRCA mortar.

## 2. Materials and Methods

### 2.1. Materials

Reference FRCA mortars, reference natural sand mortars, and FRCA mortars nanomodified with CNFs were prepared in this study. Both types of aggregates, FRCAs and natural sand, were sourced from Ozinga Concrete (Chicago, IL, USA). The characteristics of both types of aggregates (e.g., the water absorption capacity and specific gravity) were provided in prior studies [17,18]. Their X-ray diffraction patterns and chemical composition are provided in Section A of the Supplementary Materials document. The CNFs (>98% carbon basis as per the manufacturer’s specifications) were sourced from Pyrograph Products Inc. (Cedarville, OH, USA). The sourced CNFs had an average diameter of 150 nm. In addition, the CNF’s length ranged from 50 to 200 μm. Finally, the CNF’s specific surface area was 20–30 m^2^/g. Finally, the CNF composition is detailed in Table 1.

Plain natural sand mortar, FRCA mortar, and CNF-modified FRCA mortars were made in this study. In terms of the CNF mass weight fraction, as this was a preliminary study, lower percentages were initially selected to minimize material waste and better understand the making process. In addition, in prior studies [19,20], the team studied the influence of CNFs in Portland cement, using mass weight fractions of 0.1 wt.%, 0.2 wt.%, and 0.5 wt.%. The CNF series included a reference natural sand mortar (NAC-CNF-0), a reference FRCA mortar (RAC-CNF-0), and three nanomodified FRCA mortars with CNF weight percentages of 0.1 wt.%, 0.2 wt.%, and 0.5 wt.% (RAC-CNF-0.1, RAC-CNF-0.2, and RAC-CNF-0.5, respectively). Each concrete sample reinforced with fine recycled concrete aggregates was prepared using Type I cement [21]. The water-to-cement ratio was 0.44. The fine aggregate-to-cement ratio was 2.63.

### 2.2. Sample Preparation

**Figure 1:**
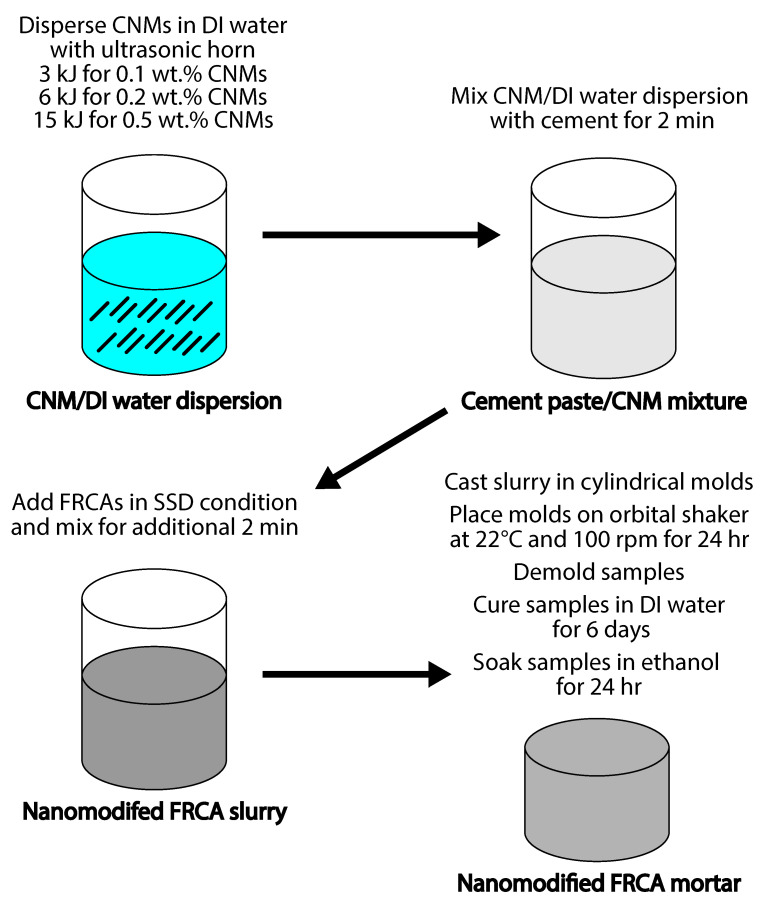
Processing method for preparation of FRCA mortars with carbon nanomaterials; CNM means carbon nanomaterial; DI is deionized water; FRCA represents fine recycled concrete aggregate; SSD means saturated surface-dry.

The innovative approach for fabricating nanomodified FRCA mortars is illustrated in Figure 1. In this method, carbon nanomaterials (CNFs) were initially dispersed in deionized water. Deionized water was used to promote CNF dispersion. CNF dispersion is affected by the presence of background ions in the water, and the ionic strength increases CNMs’ aggregation [22]. The Sonics VCX-750 ultrasonic horn (Sonics & Materials Inc., Newton, CT, USA) was employed to disperse the CNFs. The dispersion energy *E* was linear: E=30n, where n=0.1,0.2,0.5 is the CNF weight fraction in wt.%, and *E* is the dispersion energy in kJ. Subsequently, cement was incorporated into the carbon nanomaterial/water dispersion. An IKA RW 20 overhead stirrer (IKA Works Inc., Wilmington, NC, USA) was employed to further mix the dispersion. Following this, FRCAs in a saturated surface-dry state were introduced into the carbon nanomaterial/cement paste mixture. Additional mixing with the overhead stirrer ensued for two more minutes.

### 2.3. Mineralogical Composition

This study used X-ray diffraction (XRD) to resolve the chemistry of the mortars. Before the test and analysis, fragments of each mortar sample were manually crushed and ground into a fine powder capable of passing through a 400 μm sieve. The powders were further milled with ethanol using a McCrone mill, reducing the particle size to approximately 1 μm. XRD measurements were performed with a Rigaku Ultima diffractometer. In addition, the XRD equipment was configured with Bragg–Brentano geometry. As for the XRD test parameters, the energy was 40 keV. The current was 35 mA. The Bragg angle range was 2θ = 5–80°. The scan speed was 5 s/step. Finally, the step size was 0.015°. The identification of crystalline phases was performed using MDI JADE® software.

### 2.4. Microstructural Analysis

Scanning electron microscopy (SEM) was performed to resolve the multiscale structure of all mortars. The focus was on capturing the microstructure and chemistry in a non-destructive manner using non-coated samples. SEM images were collected in the backscattered mode with an FEI Quanta 650 scanning electron microscope. Imaging was conducted in the low-vacuum mode to preserve the microstructure. The chamber pressure was 0.83 torr. The accelerating voltage was 15 kV. The spot size was 4.0. These parameters were chosen to minimize the impact of the electron beam. The selected working distance was 10 mm for optimal resolution.

Backscattered environmental scanning electron microscopy (BESEM) integrated with digital image analysis has been commonly used to study cement chemistry, hydration, and porosity [23,24,25]. This methodology was employed here to investigate the influence of CNFs on concrete made with fine recycled concrete aggregates. To yield a comprehensive view of the microstructure, the magnification level chosen was 100×. A panorama approach was selected, and a large-scale view of the microstructure, covering approximately 124 mm^2^, was created by stitching together 100 SEM images taken at 100× magnification using the ImageJ software [26]. Examples of individual 100× SEM images are displayed in Section B of the Supplementary Materials document. The resolution of each 100× SEM image was 1.36 μm per pixel. The grayscale histograms of these stitched images were analyzed using a Gaussian mixture model and a digital image processing routine in Python [27]. The goal was to quantify the amount of microstructural phases. The expected microphases were pores (with a diameter above 1.36 μm), C-S-H, and aggregates. Moreover, EDS was performed on polished samples to analyze the chemical elemental composition. For SEM-EDS analysis, the parameters were the same, except for the spot size, which was increased to 5.0.

### 2.5. Permeability Analysis

The pore structure was studied using mercury intrusion porosimetry (MIP). Here is the sample preparation procedure: First, mortar samples were cold-mounted in epoxy resin with 63 mm round cylindrical molds. Next, using an Allied High Tech Techcut 4 diamond saw, four prismatic pieces were cut from each embedded sample to yield a total mass of 5–6 g for MIP testing. Then, the cut samples were dried at 5 °C in a Quincy convection laboratory oven for 24 h. MIP testing was performed with Micromeritics Autopore V equipment. The equipment can probe pore diameters ranging from 5.6 nm to 351 μm. The MIP testing took place in two steps. First, there was a low-pressure analysis with pressures in the range of 0 kPa to 290 kPa. Second, there was a high-pressure analysis with pressures in the range of 290 kPa to 228 MPa.

The pore volume measurements from the MIP testing were used to calculate the intrinsic permeability and water permeability of the mortars. The Katz–Thompson equation [28] was used to compute the intrinsic permeability *k*:

(1) $$\begin{array}{c} k=\frac{1}{89}\;d_{m}^{2}\frac{d_{m}}{d_{c}}V_{tot}\rho_{b}\phi_{d_{m}} \end{array}$$

where $d_{m}$ in m is the pore diameter that leads to the maximum hydraulic conductance (m), $d_{c}$ is the characteristic pore diameter in m, $V_{tot}$ is the cumulative intrusion volume in mL/g, $\rho_{b}$ is the bulk density in g/cm^3^, and $\phi_{d_{m}}$ is the fractional volume of connected pore spaces involving pore diameters above $d_{m}$. Here, the intrinsic permeability *k* is in m^2^.

Another assumption in this study was that the threshold pore diameter, $d_{thr}$, was the same as $d_{c}$. To determine $d_{thr}$, first, the inflection point of the cumulative intrusion volume curve was located. The linear sections of the cumulative intrusion volume curve, before and after the inflection point, were fit using linear regression models. According to Ma [29], $d_{thr}$ is found at the intersection point of the two linear regression curves. $d_{m}$ is the pore diameter that maximizes the function $y=\left(V\left(d\right)-V\left(d_{thr}\right)\right)d^{3}$; here, V(d) is the specific intrusion volume function of the pore diameter *d*. The water permeability *K* is linearly proportional to the intrinsic permeability *k* according to

(2) $$K=k\frac{\rho_{w}g}{\eta }$$

The constants used in Equation (2) were $\rho_{w}=998$ kg/m^3^ for the water density, g=9.81 m/s^2^ for the gravity, and η=0.001 N·s/m^2^ for the water dynamic viscosity.

The methodology used in this study to predict the permeability from MIP data was validated using cement paste [16], and the results agreed with published permeability values for cement paste [30,31].

## 3. Results

**Figure 2:**
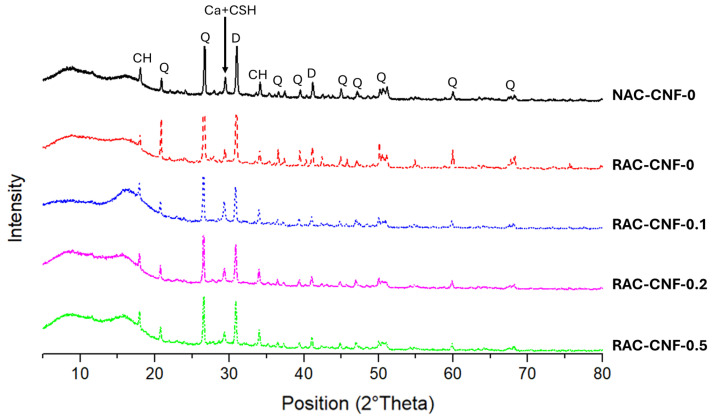
X-ray diffraction spectra for all the mortars in this study. Crystalline phases’ identification labels: Q = α-quartz; Ca = calcite; D = dolomite; CH = calcium hydroxide; Ca + CSH = calcite + CSH.

Figure 2 presents the XRD analysis of all the mortars in this study. Five mineralogical phases were present in all samples: calcite, calcium hydroxide (CH), C-S-H, dolomite, and quartz. Despite having the same phases, variations in the peak intensities highlight differences between the mortars. The primary diffraction peaks for quartz (around 21° and 27°) were more pronounced in NAC-CNF-0 compared to FRCA mortars. Additionally, the peak intensity around 29°, associated with calcite and C-S-H, was highest in RAC-CNF-0.1, followed sequentially by RAC-CNF-0, RAC-CNF-0.2, RAC-CNF-0.5, and NAC-CNF-0. Similarly, the diffraction peak near 18°, corresponding to CH, exhibited the highest intensity in RAC-CNF-0.1, followed by RAC-CNF-0.5, RAC-CNF-0.2, NAC-CNF-0, and RAC-CNF-0. The peak intensity near 34°, another indicator of CH, was the highest in RAC-CNF-0.5, with descending intensities in RAC-CNF-0.2, NAC-CNF-0, RAC-CNF-0.1, and RAC-CNF-0.

The CH orientation index *I* for each mortar was calculated to examine further how carbon nanomaterials influence the intensities of the diffraction peaks corresponding to CH [32]:

(3) $$\begin{array}{c} I=\frac{1}{0.74}\frac{{\mathrm{CH}}_{\left(001\right)}}{{\mathrm{CH}}_{\left(101\right)}} \end{array}$$

where ${\mathrm{CH}}_{\left(001\right)}$ is the CH peak intensity in the (001) direction and ${\mathrm{CH}}_{\left(101\right)}$ is the CH peak intensity in the (101) direction. The values of the CH peak intensity in the (001) and (101) directions are given in Section C of the Supplementary Materials document. *I* was equal to 2.232, 2.217, 2.896, 2.256, and 2.337 for NAC-CNF-0, RAC-CNF-0, RAC-CNF-0.1, RAC-CNF-0.2, and RAC-CNF-0.5, respectively. In addition, an increase in the CH intensities for both directions (001) and (101) was observed for CNF fractions of 0.2 wt.% and 0.5 wt.%, suggesting an increase in the CH mass fraction. These results suggest that CNFs increase the CH orientation index of concrete made with fine recycled concrete aggregates. These findings show that CNFs affect the distribution of C-S-H products within RAC.

### 3.1. Microstructural Observation

#### 3.1.1. SEM and Digital Image Analysis

**Figure 3:**
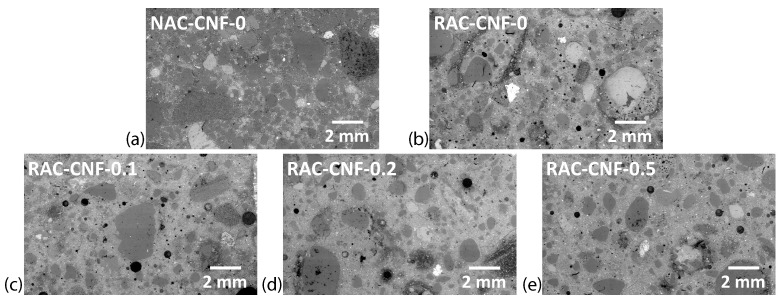
Stitched SEM images (at a magnification of 100×) of (**a**) NAC-CNF-0, (**b**) RAC-CNF-0, (**c**) RAC-CNF-0.1, (**d**) RAC-CNF-0.2, and (**e**) RAC-CNF-0.5.

**Figure 4:**
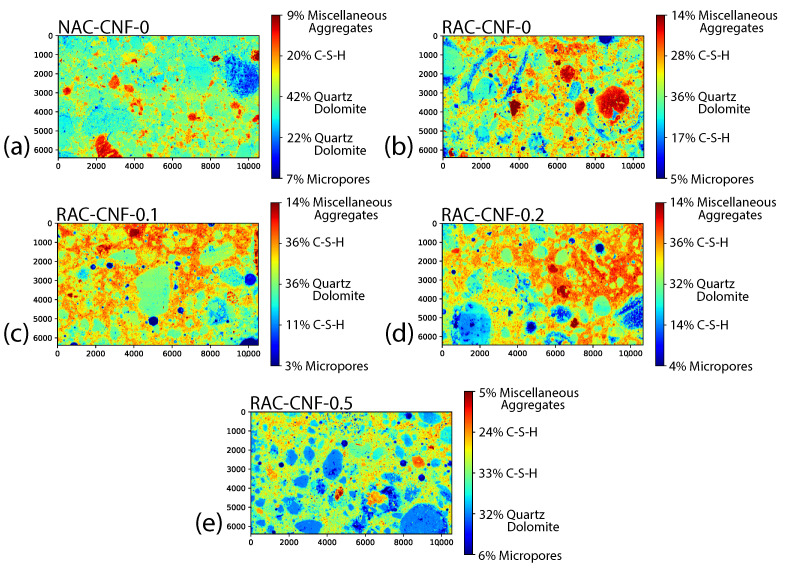
Digital image analysis of the images in Figure 3. (**a**) NAC-CNF-0, (**b**) RAC-CNF-0, (**c**) RAC-CNF-0.1, (**d**) RAC-CNF-0.2, and (**e**) RAC-CNF-0.5; C-S-H = calcium silicate hydrate matrix. Micropores indicate the fraction of pores with a diameter over 1.36 μm.

Figure 3 displays the stitched SEM images for all five mortars, with a magnification of 100×. Section D of the Supplementary Materials document displays the individual stitched SEM images. The natural sand mortar exhibited a distinct microstructure compared to FRCA mortars. A digital image analysis of Figure 3 is shown in Figure 4 to quantify the different relative amounts of microstructural phases. The two C-S-H phases refer to two C-S-H phases with distinct chemical compositions, as evidenced by distinct BESEM gray levels. For instance, the FRCA mortars had a higher relative amount of the C-S-H matrix than the reference natural sand mortar, NAC-CNF-0, which consisted largely of aggregates. Based on SEM-EDS analyses, quartz and dolomite were the primary aggregates in the mortars, while some miscellaneous aggregates (e.g., iron-based aggregates) were also identified. Additionally, with digital image analysis, RAC-CNF-0.1 and RAC-CNF-0.2 were found to have slightly less micropores (with a diameter over 1.36 μm) than the reference FRCA mortar, RAC-CNF-0. Therefore, SEM imaging indicated that CNFs reduced the pore size of CNF-modified FRCA mortars.

**Figure 5:**
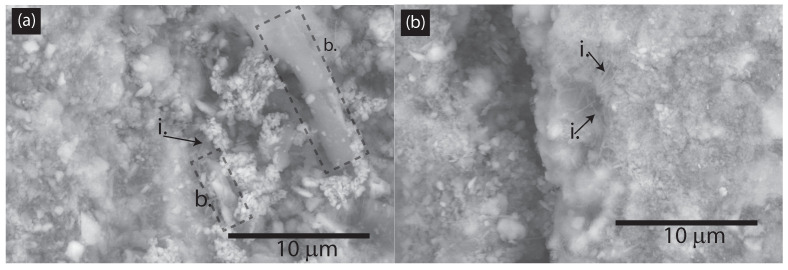
BESEM images of (**a**) RCA-CNF-0.1 and (**b**) RCA-CNF-0.5. i = individual CNF fiber. b = bundle of CNF fibers.

An important question is how to evaluate the dispersion of CNFs within the concrete matrix. Traditional ways to evaluate CNMs’ dispersion in solvents include the optical absorbance, atomic force microscopy, photoluminescence, and Zeta potential [33]; however, these methods do not predict the dispersion of CNMs within cement matrices as the environment in cement matrices is more alkaline and CNM secondary aggregation occurs frequently. Moreover, the secondary re-agglomeration of CNMs in cement matrices is common [34]. Another way to evaluate the dispersion of CNMs is via BESEM analysis, which was the approach chosen in this study. For instance, Figure 5 displays BESEM images of samples RCA-CNF-0.1 and RCA-CNF-0.5 at a magnification of 7500×. The individual BESEM images are shown in Section E of the Supplementary Materials document. In sample RCA-CNF-0.1 (cf. Figure 5a), individual CNF fibers could be seen, along with two bundles of CNF fibers. In terms of the width, the individual CNF fibers had an average width of 164 ± 33 nm, which is within the manufacturer’s specification of an average CNF diameter of 150 nm. As for the CNF bundles seen in sample RCA-CNF-0.1, the width of the first bundle was approximately 1166 nm, corresponding to 8 CNF fibers bundled; the width of the second CNF bundle was 2003 nm, corresponding to 13 NF fibers bundled. In sample RCA-CNF-0.5 (cf. Figure 5b), only individual CNF fibers could be seen, and no bundles. Therefore, BESEM analysis suggested a better dispersion of CNF fibers in sample RCA-CNF-0.5 (with a CNF mass fraction of 0.5 wt.%) than in sample RCA-CNF-0.1 (with a CNF mass fraction of 0.1 wt.%). In terms of dispersion, the difference between both samples was a longer dispersion time for sample RCA-CNF-0.5. Our analysis was limited due to the 2-D nature of SEM imaging, the local nature of our study, and the low resolution (35 nm per pixel). Nevertheless, BESEM analysis suggested a better dispersion of CNF fibers in sample RCA-CNF-0.5 than in sample RCA-CNF-0.1.

#### 3.1.2. SEM-EDS

**Figure 6:**
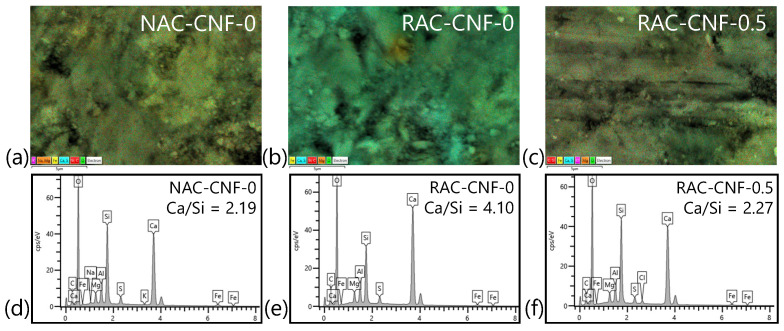
SEM images (at a magnification of 10,000×) and EDS analysis of the binder phase in (**a**) NAC-CNF-0, (**b**) RAC-CNF-0, and (**c**) RAC-CNF-0.5; corresponding EDS spectra for the SEM-EDS images of the binder phase in (**d**) NAC-CNF-0, (**e**) RAC-CNF-0, and (**f**) RAC-CNF-0.5.

With SEM-EDS, CNFs were additionally found to affect the chemistry of concrete made with fine recycled concrete aggregates. Figure 6 shows representative SEM images and EDS spectra of the binder in NAC-CNF-0, RAC-CNF-0, and RAC-CNF-0.5. For each concrete sample made with fine recycled concrete aggregates, three sites were probed. The full EDS analysis is provided in Section F of the Supplementary Materials document. From the EDS analysis, the value of the Ca/Si ratio was found to be 2.18 ± 0.04 for NAC-CNF-0, 3.92 ± 0.89 for RAC-CNF-0, and 2.35 ± 0.22 for RAC-CNF-0.5. The Ca/Si ratio of both NAC-CNF-0 and RAC-CNF-0.5, between 2 and 2.3, is characteristic of mature C-S-H gel [35]; meanwhile, the Ca/Si ratio of RAC-CNF-0, close to 3, is reminiscent of unhydrated cement alite [36]. This result suggests that 0.5 wt.% CNFs decreased the Ca/Si weight ratio of the binder phase in fine RAC, thereby accelerating cement hydration.

### 3.2. Pore Structure

The pore structure characteristics for the mortars in the CNF series are given in Table 2. Section G of the Supplementary Materials document provides more details about the methodology. The results indicate that natural sand mortar and FRCA mortars have different pore structures, and CNFs affect the pore structure of FRCA mortar. For instance, the porosity of the reference FRCA mortar, RAC-CNF-0 (28.0%), was 67.7% greater than the porosity of the reference natural sand mortar, NAC-CNF-0 (16.7%). Additionally, RAC-CNF-0 had a smaller skeletal density (2.27 g/cm^3^) than NAC-CNF-0 (2.47 g/cm^3^). RAC-CNF-0 had a higher water permeability (3.69 × 10^−11^ m/s) compared to NAC-CNF-0 (3.60 × 10^−11^ m/s). The addition of 0.1 wt.% CNFs in FRCA mortar increased the porosity by 8.2%, while additions of 0.2 wt.% and 0.5 wt.% CNFs decreased the porosity by 2.1% and 8.9%, respectively. The addition of 0.1 wt.% CNFs did not appear to affect the skeletal density, while additions of 0.2 wt.% and 0.5 wt.% CNFs resulted in slight decreases in the skeletal density. Lastly, the addition of 0.1 wt.% CNFs resulted in a 26.8% greater water permeability, while additions of 0.2 wt.% and 0.5 wt.% CNFs resulted in 22.8% and 39.3% decreases in the water permeability, respectively. The increase in porosity observed for RCA-CNF-0.1 can be attributed to the poor dispersion of CNFs and the presence of CNF bundles; see Figure 5. Thus, CNFs at additions of 0.2 wt.% and 0.5 wt.% significantly refined the pore structure of FRCA mortar. This refinement of the pore structure for CNF mass fractions of 0.2 wt.% and 0.5 wt.% can be explained by the better dispersion of CNFs and their effect on cement hydration.

**Figure 7:**
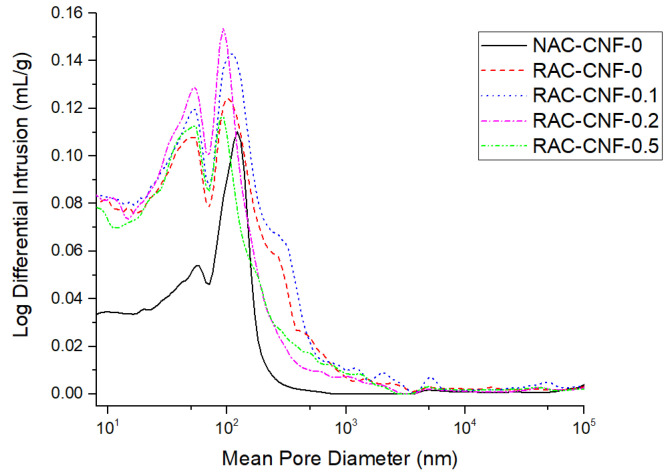
Log differential pore size distributions for the mortars in the CNF series.

Figure 7 displays the pore size distributions for all mortars, focusing on the log differential of the intrusion volume. The pore size distribution for natural sand mortar (solid black line) significantly differed from that of FRCA mortars. For the FRCA mortars, the maximum value of the intrusion volume occurred for mean pore diameters of 122 nm, 100 nm, 107 nm, 94 nm, and 94 nm for NAC-CNF-0, RAC-CNF-0, RAC-CNF-0.1, RAC-CNF-0.2, and RAC-CNF-0.5, respectively. Moreover, the maximum intrusion volume was greatest in RAC-CNF-0.2, followed by RAC-CNF-0.1, RAC-CNF-0, RAC-CNF-0.5, and NAC-CNF-0. RAC-CNF-0, RAC-CNF-0.2, and RAC-CNF-0.5 showed a second peak on the log differential intrusion volume curve at 55 nm, while NAC-CNF-0 and RAC-CNF-0.1 exhibited second peaks at 59 nm and 51 nm, respectively. The pore size distributions of RAC-CNF-0.2 and RAC-CNF-0.5 exhibited a noticeably lower intrusion volume than that of RAC-CNF-0 and RAC-CNF-0.1 at mean pore diameters between 100–1000 nm. Therefore, the porosity of FRCA mortar was reduced when FRCAs were nanomodified with CNFs at weight fractions of 0.2 wt.% and 0.5 wt.%.

## 4. Discussion

The XRD results (Figure 2) show that CNFs affect the crystal structure of FRCA mortar. Specifically, CNFs increased the intensities of the diffraction peaks corresponding to CH at 18° and 34°. Thus, CNFs boosted the growth of CH crystals in fine RAC. Prior studies have reported an increase in CH growth for CNF-nanomodified natural sand mortar [37], and that increase was linked to the altered hydration of alite. In this study, we report an increase in CH growth for CNF-nanomodified FRCA mortar.

Furthermore, the EDS results (Figure 6) show that CNFs affect the chemical composition of the binder in FRCA mortar. Specifically, 0.5 wt.% CNFs were found to decrease the calcium-to-silicon weight ratio of the binder to a level that was similar to that of the binder in natural sand mortar. The change in the calcium-to-silicon weight ratio due to CNFs aligns with Gao et al.’s results; they showed that CNFs affect the calcium-to-silicon ratio in the interfacial transition zone of plain concrete [38]. In this study, we demonstrated similar effects of CNFs on the distribution of calcium and silicon for recycled aggregate concrete.

The SEM imaging (Figure 3) and digital image analysis (Figure 4) showed that CNFs affect the microstructure of fine RAC. Specifically, the stitched SEM images at a magnification of 100× (Figure 3) indicate that adding 0.1 wt.% CNFs raised the maximum pore size, while adding 0.2 wt.% and 0.5 wt.% CNFs decreased the maximum pore size. Digital image analyses of these stitched SEM images (Figure 4) indicated that CNFs at weight fractions of 0.1 wt.% and 0.2 wt.% slightly decreased the number of micropores. In comparison, a weight fraction of 0.5 wt.% led to a moderate increase in the number of micropores. The moderate increase in micropores with 0.5 wt.% CNFs can be explained by the difficulty in homogeneously dispersing CNFs in the binder for high concentrations. This is among the few reports on how CNFs affect the maximum pore size and distribution of microstructural phases in recycled aggregate concrete.

The MIP results (Figure 7 and Table 2) show that low reinforcement levels of CNFs, 0.1 wt.%, initially coarsened the pore structure of FRCA mortar due to the poor dispersion of CNFs at 0.1 wt.%. Meanwhile, higher reinforcement levels, 0.2 wt.% and 0.5 wt.%, refined the pore size distribution of FRCA mortar, given the better dispersion of CNFs and their catalytic effect. The optimum weight percentage of CNFs to enhance the pore size distribution of FRCA mortar was 0.5 wt.%: the porosity decreased by 8.9% and the water permeability decreased by 39.3%. Regarding mechanisms, the pore structure refinement effects of 0.2 wt.% and 0.5 wt.% CNFs (i.e., the removal of pores and conversion of larger pores to smaller pores) are believed to be linked to the ability of the CNFs to serve as nanofillers that connect hydration products, fill microvoids, and modify the C-S-H distribution. From the XRD and EDS results, we found that CNFs promote the nucleation and growth of hydration products, such as calcium silicate hydrate (C-S-H) gels and calcium hydroxide (CH). This results in the densification of the cement matrix, particularly around RCAs, thereby refining the pore structure. Moreover, the high tensile strength and aspect ratio of CNFs allow them to bridge microcracks, preventing the coalescence of pores into larger voids. This leads to a more uniform and refined pore size distribution. Finally, CNFs improve the connectivity and packing density of hydration products, converting larger pores into smaller ones. The observed CNF-induced refinement of the pore size distribution agrees with Wang [13]’s findings; they showed that CNFs at additions of up to 0.5 wt.% decreased the porosity and relative permeability coefficient of conventional concrete with a water-to-cement ratio of 0.36 [13]. However, our report is among the very few reports to provide data on the pore structure refinement effects of CNFs in RAC.

Scaling up CNF-modified recycled aggregate concrete (RAC) for real-world applications presents notable challenges but also offers transformative potential. A primary issue is the high cost of carbon nanofibers, which could substantially increase production expenses, potentially hindering widespread adoption. Achieving the consistent dispersion of CNFs in large-scale production is another significant challenge. Yet, the advantages of CNF-modified RAC are compelling. By significantly improving the durability, and microstructural properties of RAC, CNFs make recycled aggregates a viable and sustainable alternative to natural aggregates. The enhanced porosity and permeability characteristics also make CNF-modified RAC particularly suitable for applications in aggressive environments, such as those involving chemical exposure and freeze–thaw cycles. To enable successful scaling, further research and innovation are required. Efforts should focus on reducing the cost of CNFs, refining dispersion and mixing techniques, and ensuring that environmental and occupational safety standards are met. Establishing comprehensive regulatory frameworks will be essential to build confidence in using CNFs and facilitate their integration into industry practices. With these measures, CNF-modified RAC could play a pivotal role in advancing sustainable and high-performance construction solutions.

Future research will characterize the mechanical properties of the CNF-modified RAC samples, optimize CNF dispersion techniques, reduce material costs, and evaluate the long-term performance in real-world applications. The incorporation of nanotechnology into RAC has the potential to redefine its role in sustainable construction, offering a viable solution for a wide range of structural and environmental applications [5,6,7,8,13].

## 5. Conclusions

We developed an innovative process method to fabricate FRCA mortars enhanced with carbon nanomaterials, specifically CNFs, at weight fractions of 0.1 wt.%, 0.2 wt.%, and 0.5 wt.%. This effort was guided by an interdisciplinary approach that combined analytical characterization techniques to deepen our understanding of the mechanisms by which carbon nanomaterials influence the properties of FRCA mortar across multiple scales.

- XRD analysis showed that CNFs increased the presence of calcium hydroxide (CH) within RAC.
- The CNFs increased the CH orientation index, indicating improved crystallinity and matrix reinforcement.
- SEM showed significant microstructural changes, with CNFs reducing the maximum pore size and promoting C-S-H gel formation.
- SEM-EDS showed a reduced Ca/Si ratio with 0.5 wt.% CNFs, suggesting that CNFs accelerate cement hydration within RAC.
- MIP showed that CNFs refined the pore structure and reduced the porosity, with the highest reduction in porosity, 8.9%, observed at a reinforcement level of 0.5 wt.%.
- MIP showed that CNFs reduced the water permeability, with the highest reduction in water permeability, 37.9%, observed at a reinforcement level of 0.5 wt.%.

Therefore, this study has demonstrated the potential of CNFs to densify RAC, promote and accelerate C-S-H gel formation, refine the pore structure, and reduce both the porosity and water permeability. This study has demonstrated the potential of CNFs to enhance the durability and performance of RAC, making it suitable for modern construction applications. In this manner, CNF nanomodification enhances the durability and performance of RAC. These findings pave the way toward sustainable and high-performance nanomodified RAC.

## Figures and Tables

**Table 1:** Mix design for CNF-modified mortars.

Sample	CNF wt.%	CNFs (g)	Water (g)	Cement (g)	FRCAs (g)	Sand (g)
NAC-CNF-0	0	0	10.81	24.56	-	64.63
RAC-CNF-0	0	0	10.81	24.56	64.63	-
RAC-CNF-0.1	0.1	0.025	10.81	24.56	64.63	-
RAC-CNF-0.2	0.2	0.05	10.81	24.56	64.63	-
RAC-CNF-0.5	0.5	0.12	10.81	24.56	64.63	-

**Table 2:** Measured pore structure parameters for the mortars in this study; $d_{thr}$ is the threshold pore diameter, *k* is the intrinsic permeability, and *K* is the water permeability.

Sample	Porosity	Skeletal Density	$d_{\mathrm{thr}}$	*k*	*K* at 20 °C
	(%)	(g/cm^3^)	(nm)	(m^2^)	(m/s)
NAC-CNF-0	16.7	2.47	164	$3.68\times 10^{-18}$	$3.60\times 10^{-11}$
RAC-CNF-0	28.0	2.27	193	$3.77\times 10^{-18}$	$3.69\times 10^{-11}$
RAC-CNF-0.1	30.3	2.27	203	$3.77\times 10^{-18}$	$3.69\times 10^{-11}$
RAC-CNF-0.2	27.4	2.25	150	$2.91\times 10^{-18}$	$2.85\times 10^{-11}$
RAC-CNF-0.5	25.5	2.24	155	$2.29\times 10^{-18}$	$2.29\times 10^{-11}$

## Data Availability

The original contributions presented in this study are included in the article/Supplementary Materials. Further inquiries can be directed to the corresponding author.

## Supplementary Materials

The following supporting information can be downloaded at: https://www.mdpi.com/article/10.3390/nano15040253/s1, Table S1: Chemical composition of natural sand. D = dolomite. M = minrecordite. Q = quartz. Figure S1: X-ray diffraction pattern of natural sand. Figure S2: X-ray diffraction pattern of fine recycled concrete aggregates; Table S2: Chemical composition of fine recycled concrete aggregates. C = calcite. D = dolomite. P = portlandite. Q = quartz. S = samarium; Figure S3: SEM image of NAC-CNF-0 at magnification 100×; Figure S4: SEM image of RAC-CNF-0 at magnification 100×. Figure S5: SEM image of RAC-CNF-0.1 at magnification 100×; Figure S6: SEM image of RAC-CNF-0.2 at magnification 100×; Figure S7: SEM image of RAC-CNF-0.5 at magnification 100×; Table S3: The peak intensities of calcium hydroxide (C-H) in the (001) and (101) directions and the orientation index *I* for all mortars. Figure S8: SEM map NAC-CNF-0 spanning an area of 124 mm^2^; Figure S9: SEM map RAC-CNF-0 spanning an area of 124 mm^2^; Figure S10: SEM map RAC-CNF-0.1 spanning an area of 124 mm^2^; Figure S11: SEM map RAC-CNF-0.2 spanning an area of 124 mm^2^. Figure S12: SEM map RAC-CNF-0.5 spanning an area of 124 mm^2^; Figure S13: BESEM image of sample RAC-CNF-0.1 showing both individual CNF fibers and CNF bundles; Figure S14: BESEM image of sample RAC-CNF-0.1 showing individual CNF fibers. Figure S15: SEM image and EDS spectra of binder matrix in NAC-CNF-0. Site 1; Figure S16: SEM image and EDS spectra of binder matrix in NAC-CNF-0. Site 2; Figure S17: SEM image and EDS spectra of binder matrix in NAC-CNF-0. Site 3; Figure S18: SEM image and EDS spectra of binder matrix in RAC-CNF-0. Site 1; Figure S19: SEM image and EDS spectra of binder matrix in RAC-CNF-0. Site 2; Figure S20: SEM image and EDS spectra of binder matrix in RAC-CNF-0. Site 3; Figure S21: SEM image and EDS spectra of binder matrix in RAC-CNF-0.5. Site 1; Figure S22: SEM image and EDS spectra of binder matrix in RAC-CNF-0.5. Site 2; Figure S23: SEM image and EDS spectra of binder matrix in RAC-CNF-0.5. Site 3; Figure S24: Cumulative pore size distributions for all mortars. Figure S25: Determination of the threshold pore diameter for cement paste with a w/c ratio of 0.44 after 1 day of curing at room temperature and 6 days of curing in lime water; CP = cement paste; d = mean pore diameter; $d_{thr}$ = threshold pore diameter; Figure S26: Determination of the pore diameter corresponding to the maximum hydraulic conductance, dmax, for cement paste with a w/c ratio of 0.44 after 1 day of curing at room temperature and 6 days of curing in lime water; *I* = specific intrusion volume at pore diameter *d*; $I_{thr}$ = specific intrusion volume at the threshold pore diameter $d_{thr}$. Table S4: Pore structure characteristics of cement paste with a w/c ratio of 0.44 after 1 day of curing at room temperature and 6 days of curing in lime water; $d_{thr}$ = threshold pore diameter; *k* = intrinsic permeability; *K* = water permeability; Figure S27: Determination of the threshold pore diameter, $d_{thr}$, for (a) NAC-CNF-0 (b) RAC- CNF-0 (c) RAC-CNF-0.1 (d) RAC-CNF-0.2 and (e) RAC-CNF-0.5; *d* = mean pore diameter; $d_{thr}$ = threshold pore diameter; Figure S28: Determination of the threshold pore diameter, $d_{thr}$, for (a) NAC-CNT-0 (b) RAC- CNT-0 (c) RAC-CNT-0.1 (d) RAC-CNT-0.2 and (e) RAC-CNT-0.5; d = mean pore diameter; $d_{thr}$ = threshold pore diameter; Figure S29: Determination of the pore diameter corresponding to the maximum hydraulic conductance, dmax, for (a) NAC-CNF-0 (b) RAC-CNF-0 (c) RAC-CNF-0.1 (d) RAC-CNF-0.2 and (e) RAC-CNF-0.5; *I* = specific intrusion volume at pore diameter *d*; $I_{thr}$ = specific intrusion volume at the threshold pore diameter $d_{thr}$.
