# Supplementary material for: The Effect of Carbon Nanofibers on the Microstructure, Chemistry, and Pore Structure of Concrete Made with Fine Recycled Concrete Aggregates
Source: Nanomaterials (Basel). 2025 Feb 7;15(4):253. doi: 10.3390/nano15040253 (PMC11858078; doi:10.3390/nano15040253)
Supplement: Supplementary file 1 [file nanomaterials-15-00253-s001.zip › nanomaterials-3415031-supplementary.pdf]

Supplementary Material

A. Chemical Composition of Natural Sand and Fine Recycled Aggregates

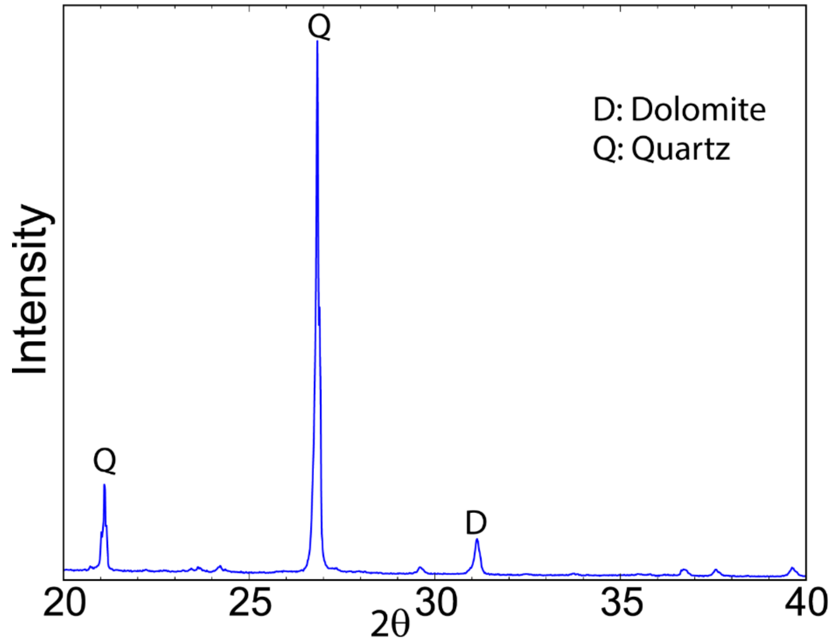

Figure S1: X-ray diffraction pattern of natural sand.

Phase	D	M	Q
Mass fraction (wt%)	12.4	7.1	80.5

Table S1: Chemical composition of natural sand. D= dolomite. M=minrecordite. Q=quartz.

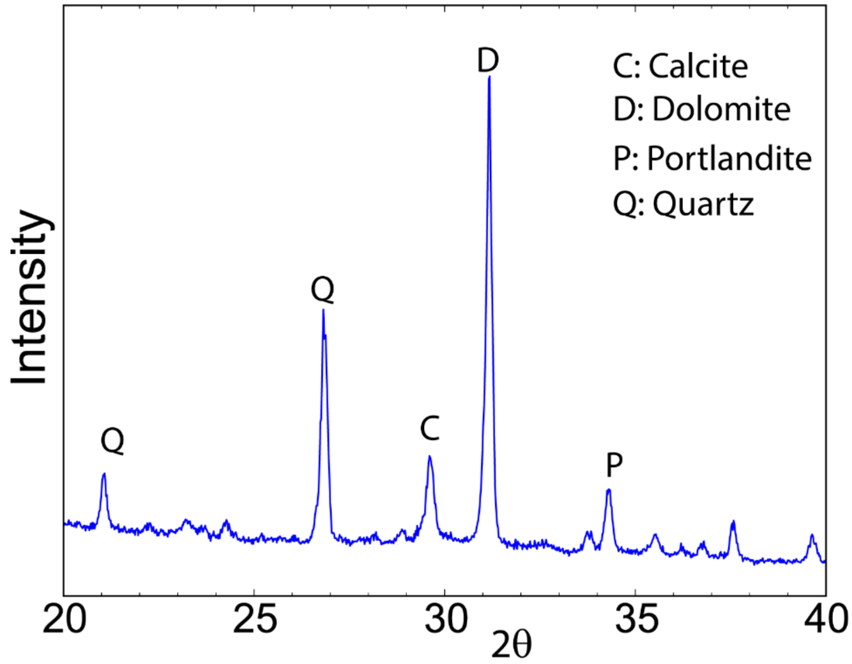

Figure S2: X-ray diffraction pattern of fine recycled concrete aggregates.

Phase	C	D	P	Q	S
Mass fraction (wt%)	12.1	52.0	9.7	23.0	12.4

Table S2: Chemical composition of fine recycled concrete aggregates. C=calcite. D= dolomite. P=portlandite. Q=quartz. S=samarium.

B. Scanning Electron Microscopy

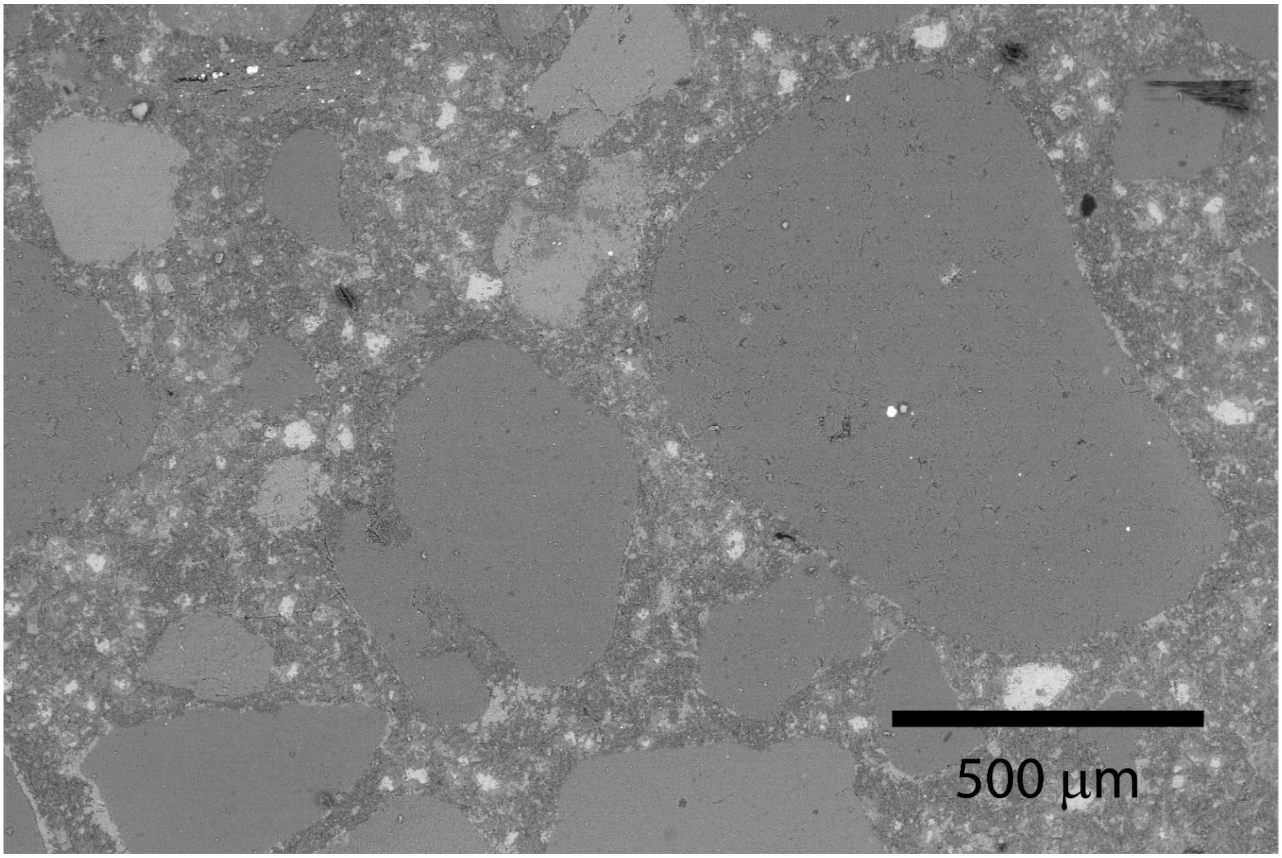

Figure S3: SEM image of NAC-CNF-0 at magnification 100×.

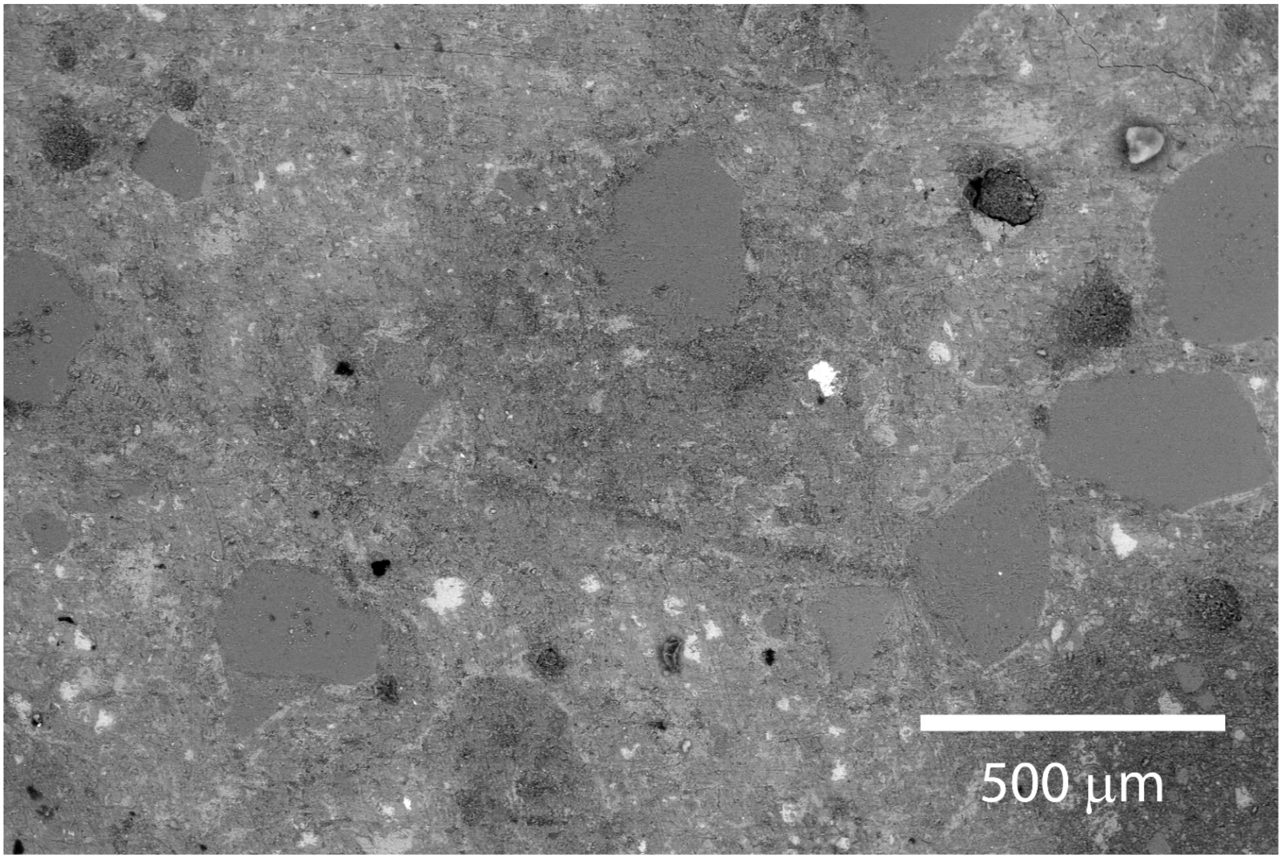

Figure S4: SEM image of RAC-CNF-0 at magnification 100×.

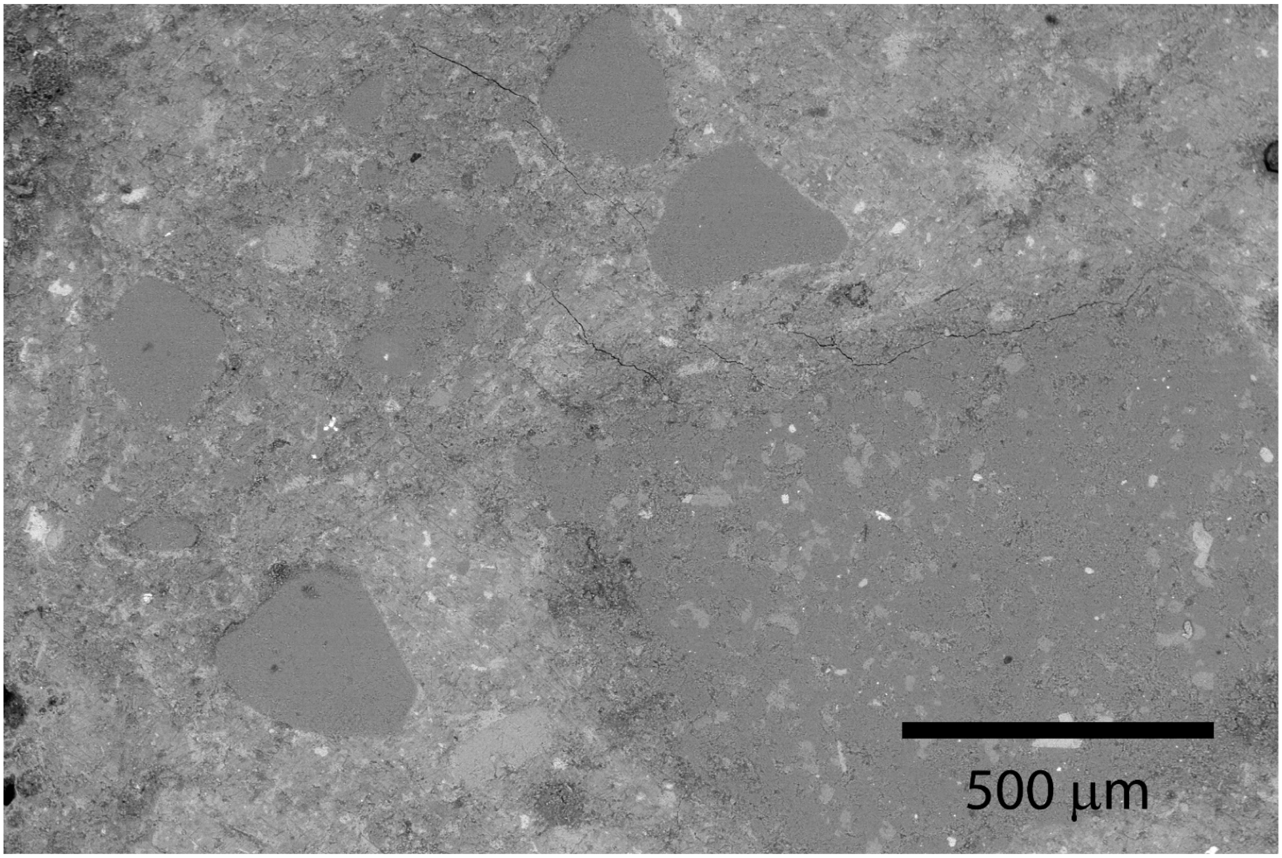

Figure S5: SEM image of RAC-CNF-0.1 at magnification 100×.

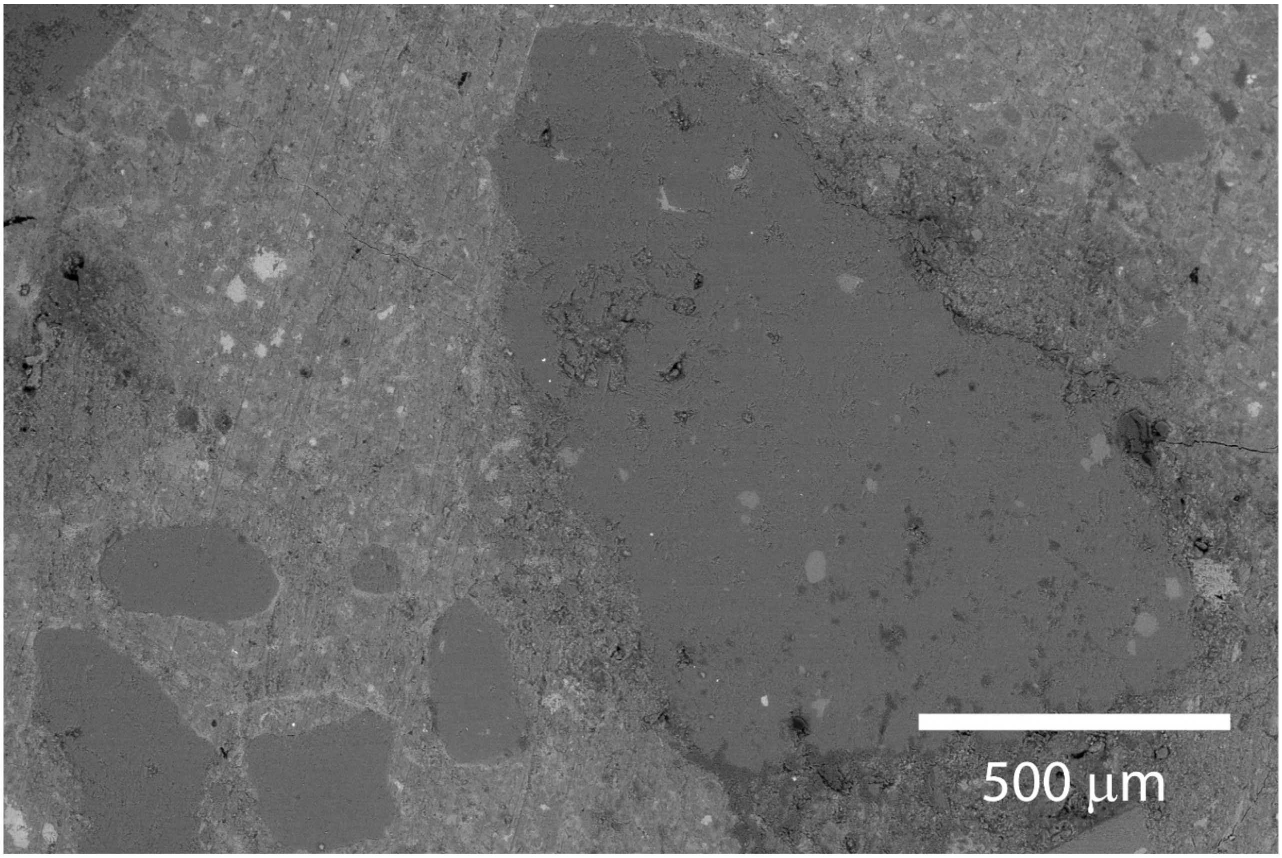

Figure S6: SEM image of RAC-CNF-0.2 at magnification 100×.

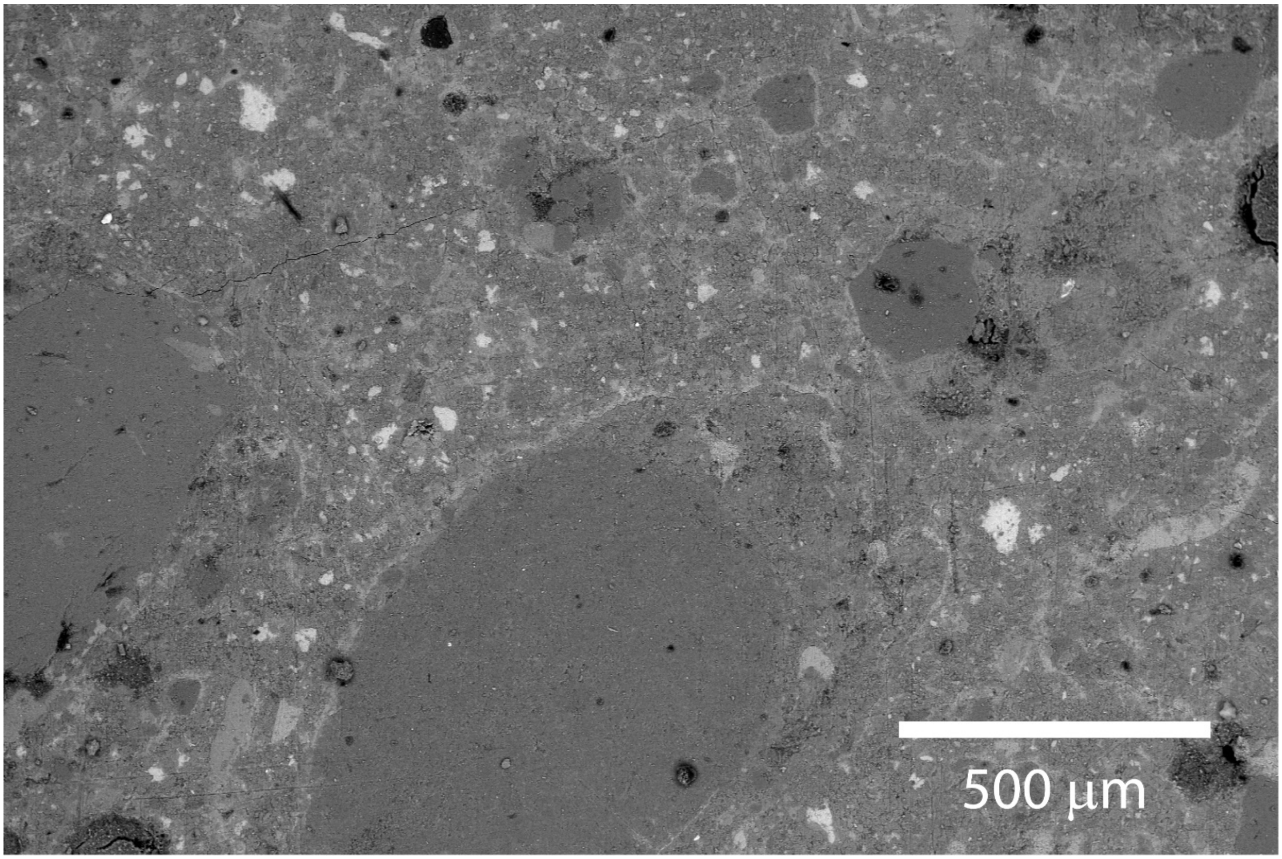

Figure S7: SEM image of RAC-CNF-0.5 at magnification 100×.

C. X-Ray Diffraction Analysis

Table S3: The peak intensities of calcium hydroxide (C-H) in the (001) and (101) directions and the orientation index *I* for all mortars.

Sample	C-H Peak Intensity in (001) Direction	C-H Peak Intensity in (101) Direction	Orientation Index *I*
NAC-CNF-0	2636	1596	2.232
RAC-CNF-0	2535	1545	2.217
RAC-CNF-0.1	3399	1586	2.896
RAC-CNF-0.2	2740	1641	2.256
RAC-CNF-0.5	3004	1737	2.337

D. SEM Maps

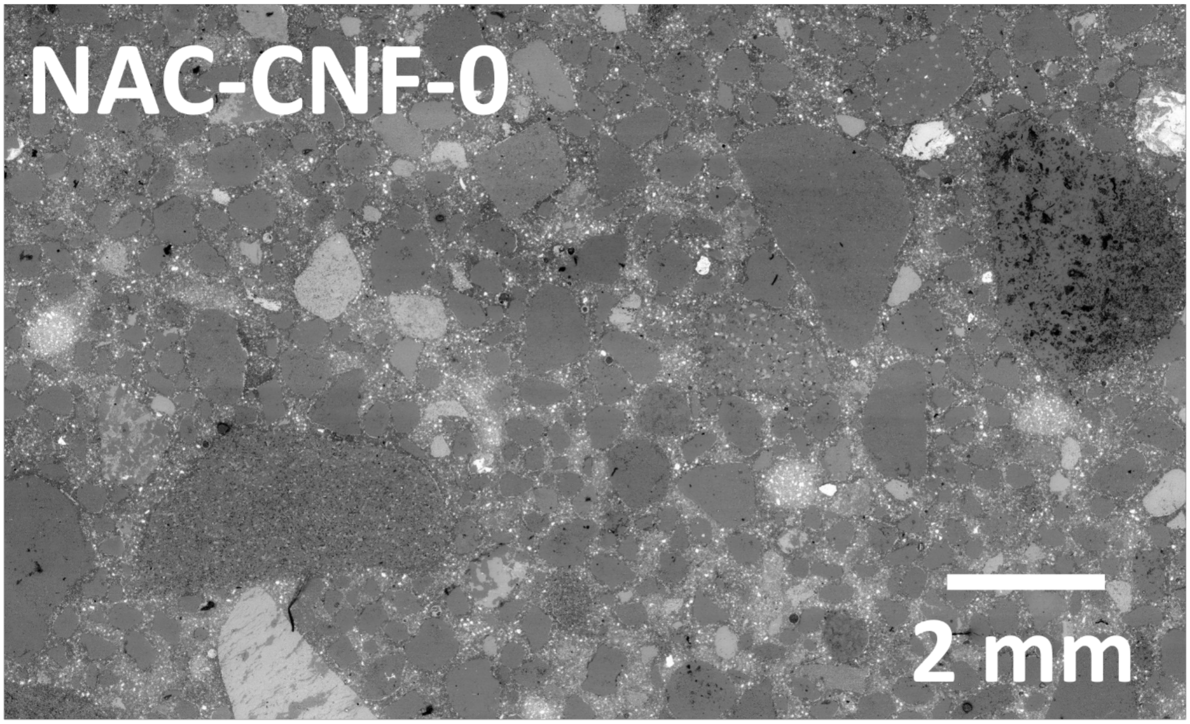

Figure S8: SEM map NAC-CNF-0 spanning an area of 124 mm^2.

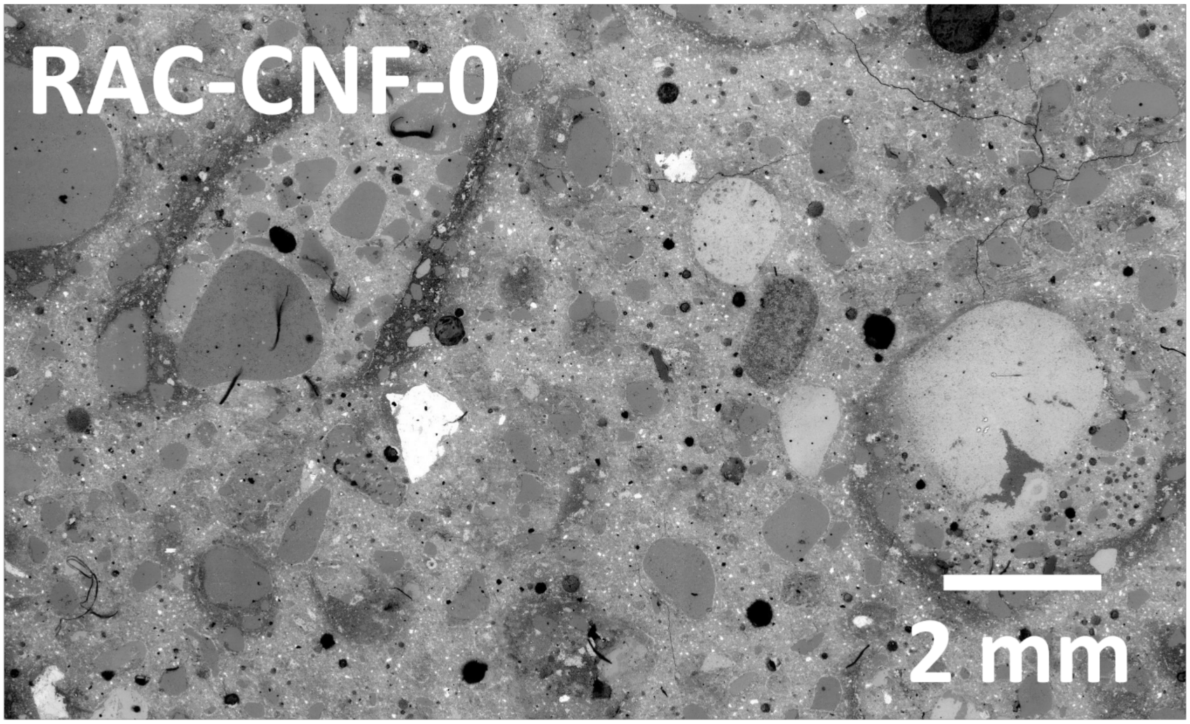

Figure S9: SEM map RAC-CNF-0 spanning an area of 124 mm^2.

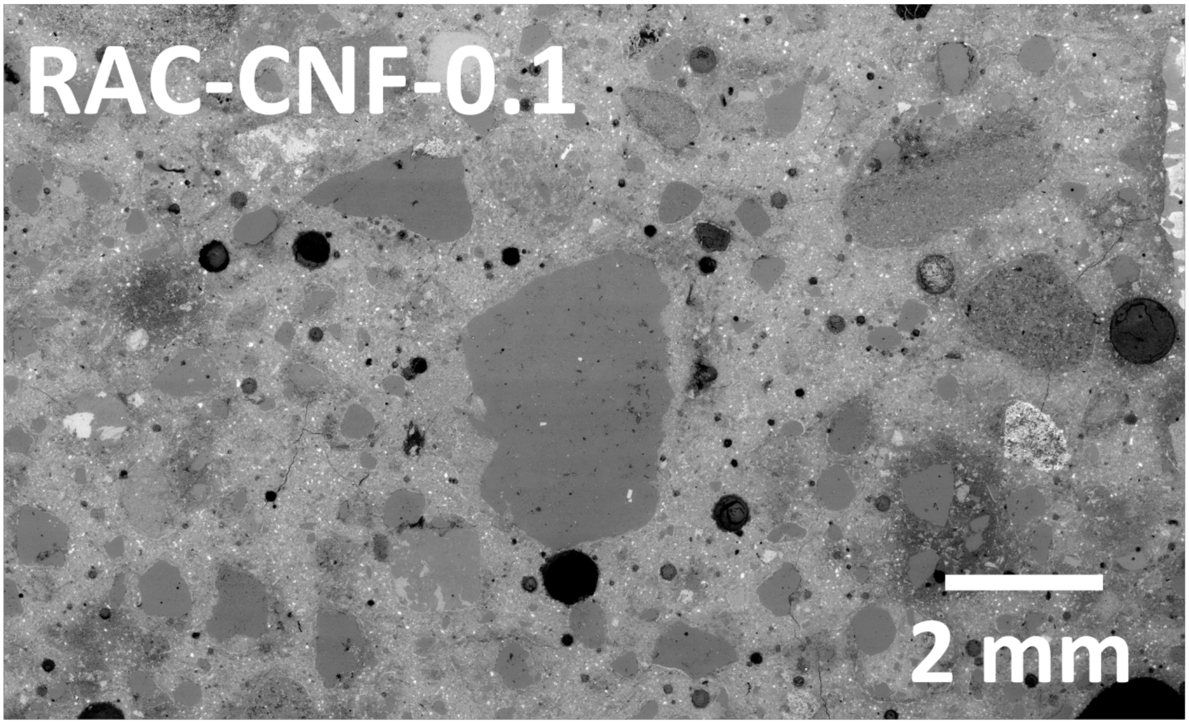

Figure S10: SEM map RAC-CNF-0.1 spanning an area of 124 mm^2.

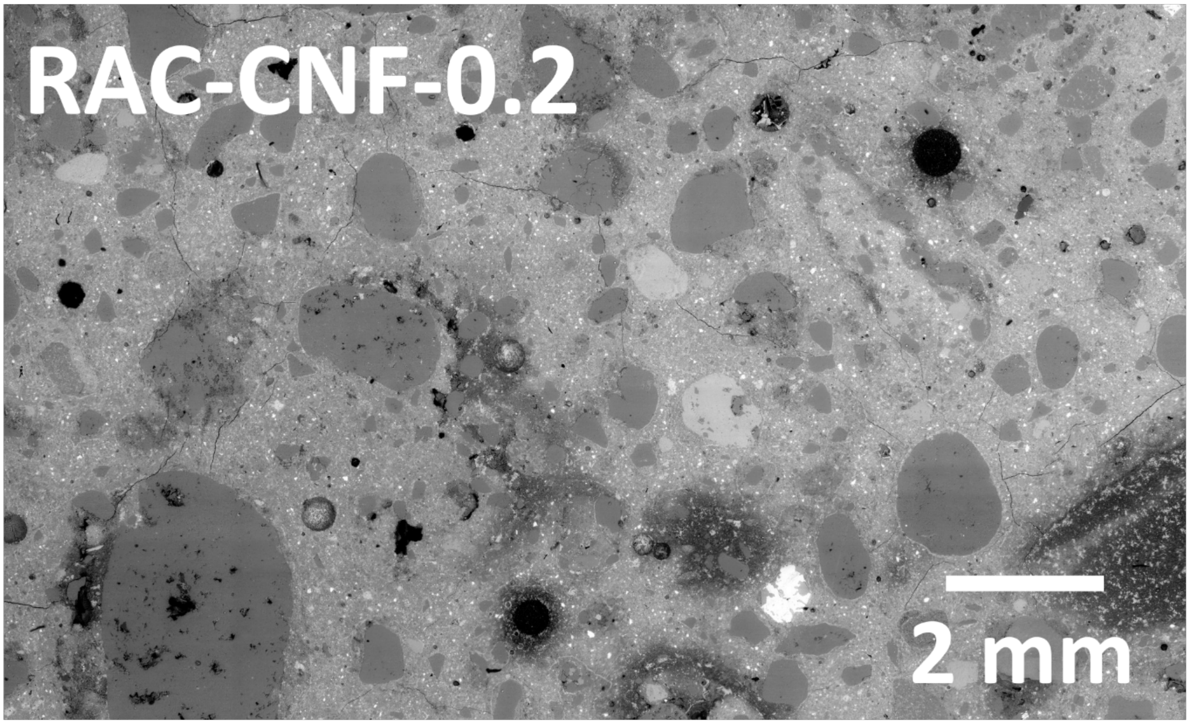

Figure S11: SEM map RAC-CNF-0.2 spanning an area of 124 mm^2.

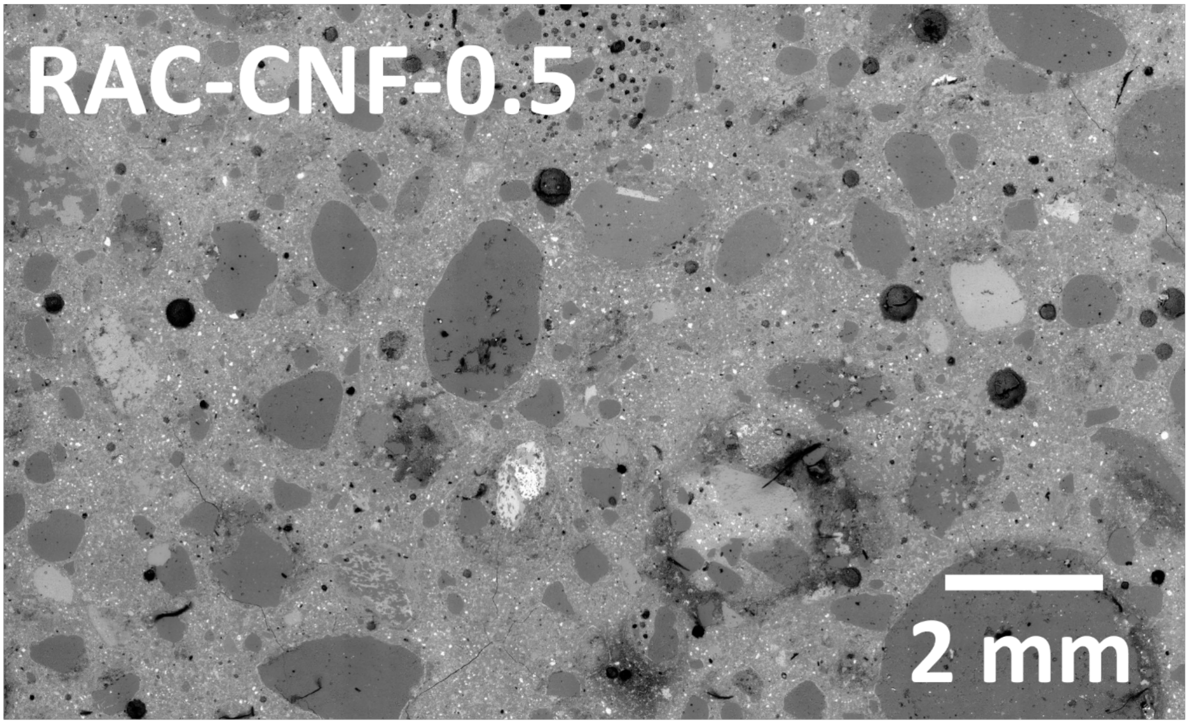

Figure S12: SEM map RAC-CNF-0.5 spanning an area of 124 mm^2.

E. BESEM images showing CNFs

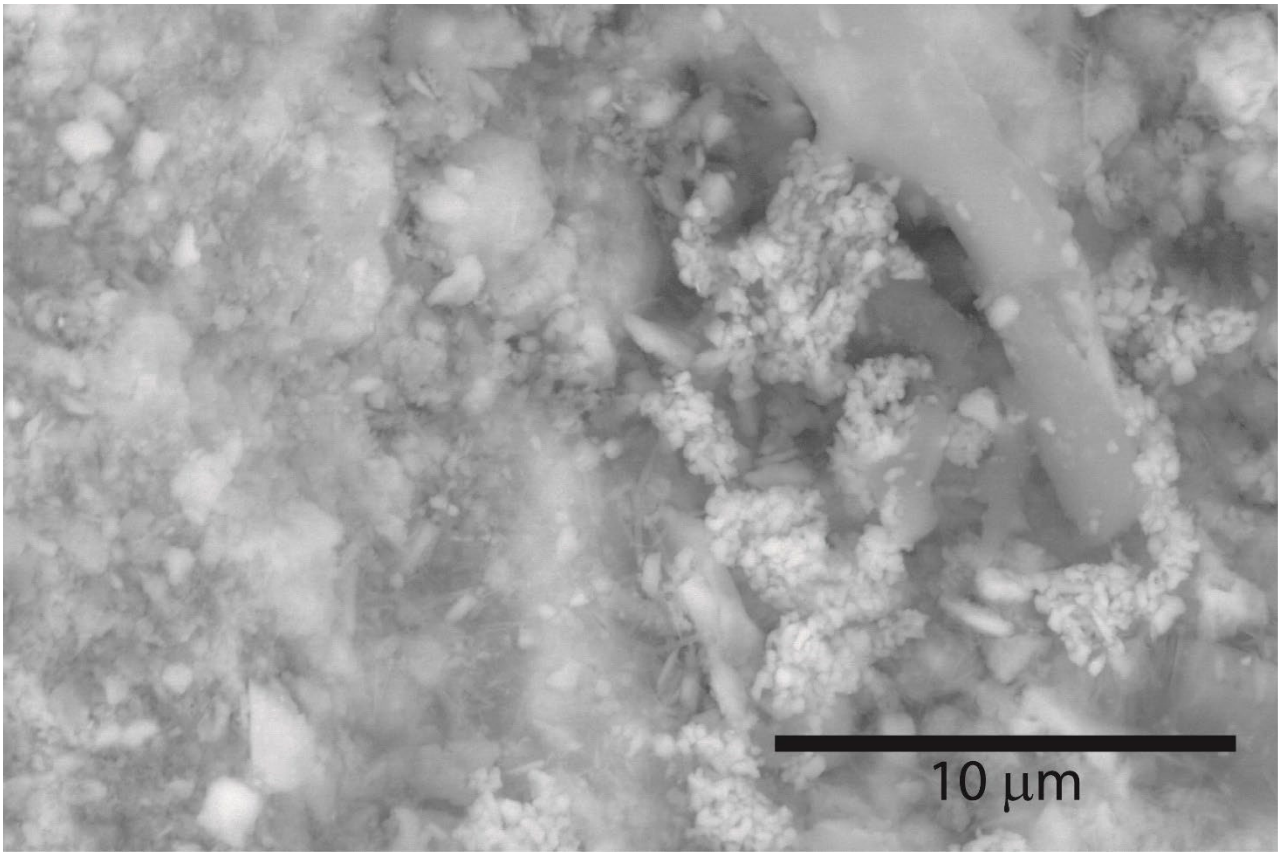

Figure S13: BESEM image of sample RAC-CNF-0.1 showing both individual CNF fibers and CNF bundles.

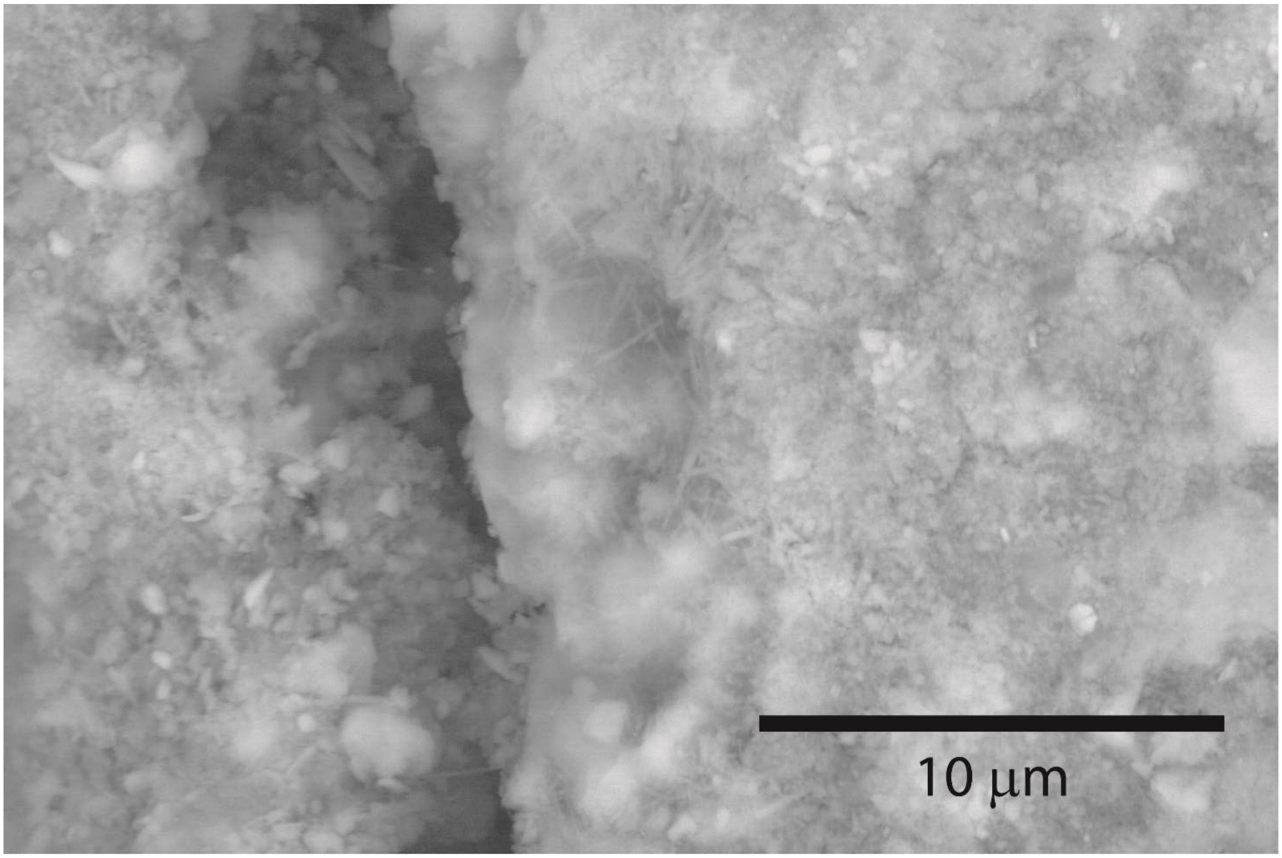

Figure S14: BESEM image of sample RAC-CNF-0.1 showing individual CNF fibers.

F. Scanning Electron Microscopy /Electron Dispersive Spectroscopy

Sample NAC-CNF-0

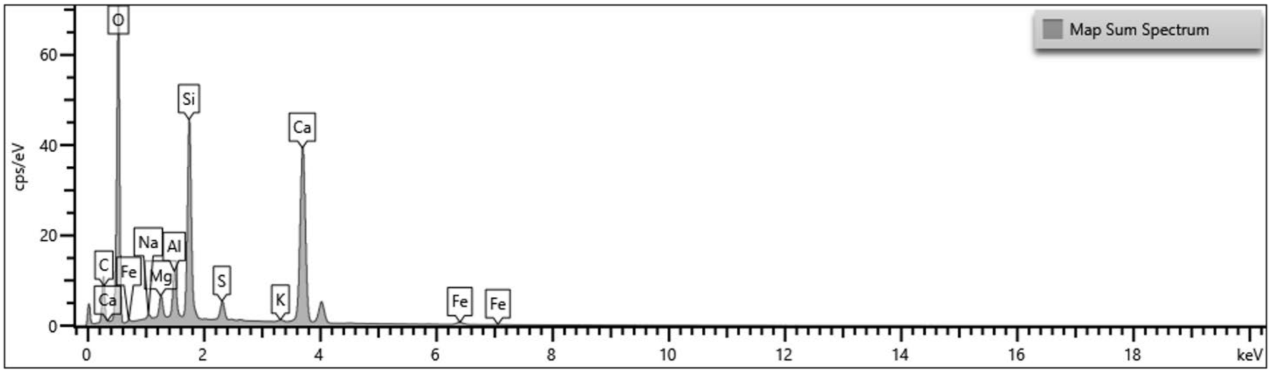

Map Sum Spectrum		
Element	Wt%	Wt% Sigma
O	52.15	0.07
Na	0.32	0.01
Mg	1.46	0.01
Al	2.85	0.02
Si	12.78	0.03
S	1.54	0.01
K	0.26	0.01
Ca	27.32	0.05
Fe	1.33	0.03
Total:	100	

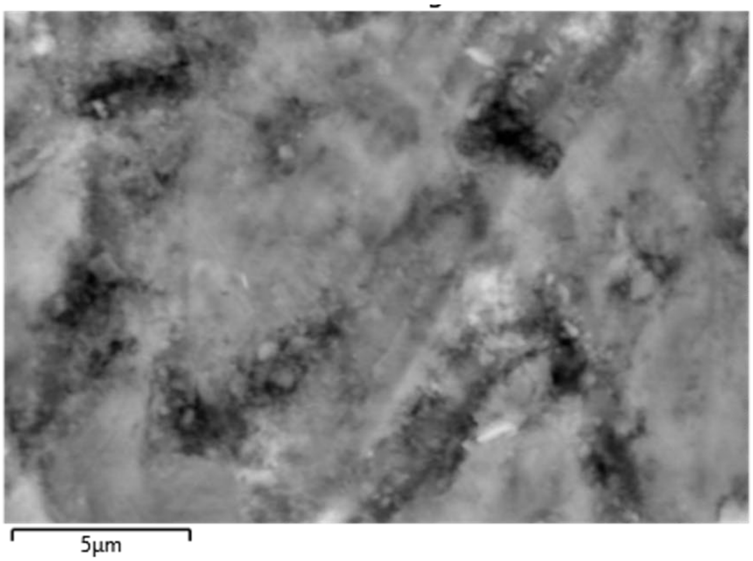

Figure S15: SEM image and EDS spectra of binder matrix in NAC-CNF-0. Site 1.

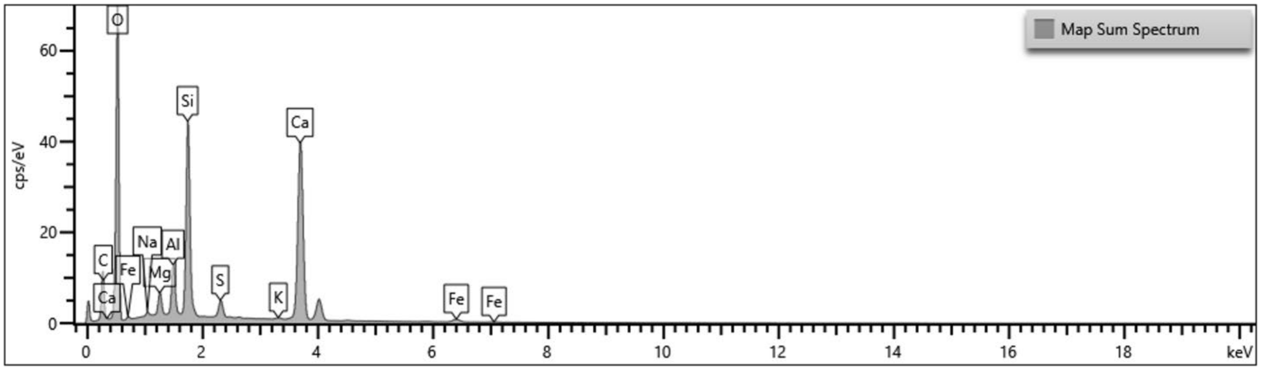

Map Sum Spectrum		
Element	Wt%	Wt% Sigma
O	51.72	0.07
Na	0.29	0.01
Mg	1.5	0.01
Al	3.13	0.02
Si	12.49	0.03
S	1.41	0.01
K	0.21	0.01
Ca	27.56	0.05
Fe	1.69	0.04
Total:	100	

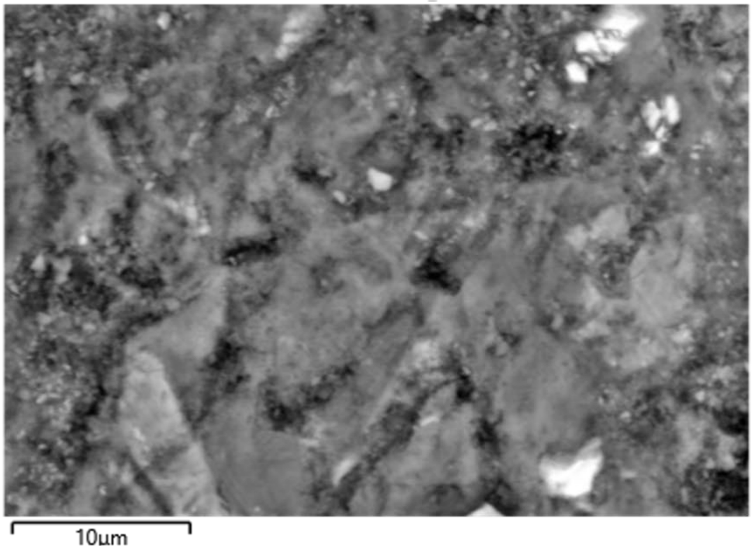

Figure S16: SEM image and EDS spectra of binder matrix in NAC-CNF-0. Site 2.

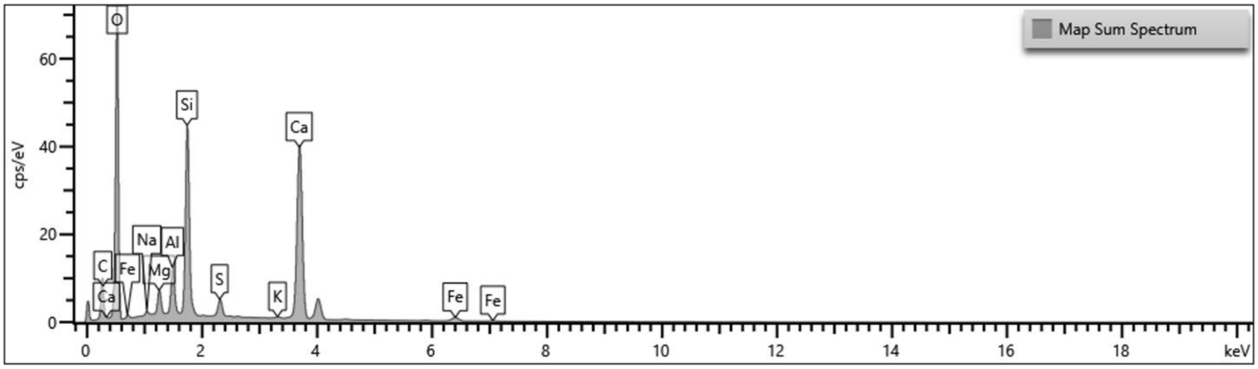

Map Sum Spectrum		
Element	Wt%	Wt% Sigma
O	51.74	0.07
Na	0.27	0.01
Mg	1.63	0.01
Al	2.92	0.02
Si	12.36	0.03
S	1.44	0.01
K	0.2	0.01
Ca	27.25	0.05
Fe	2.19	0.04
Total:	100	

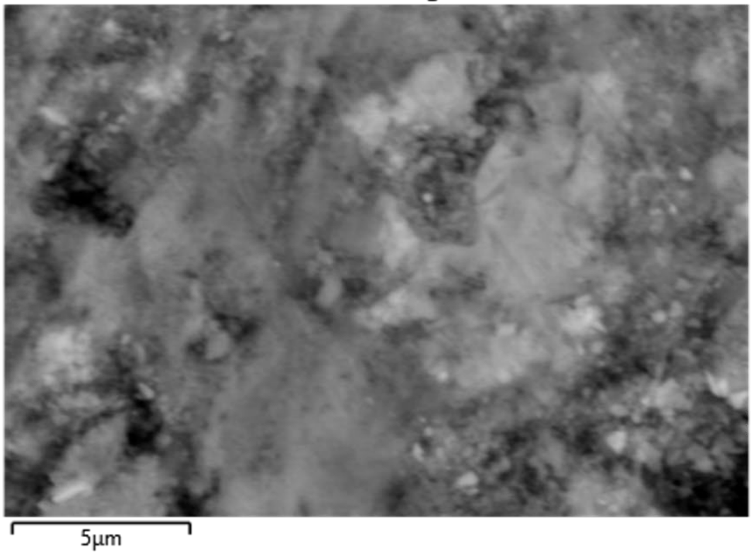

Figure S17: SEM image and EDS spectra of binder matrix in NAC-CNF-0. Site 3.

Sample RAC-CNF-0

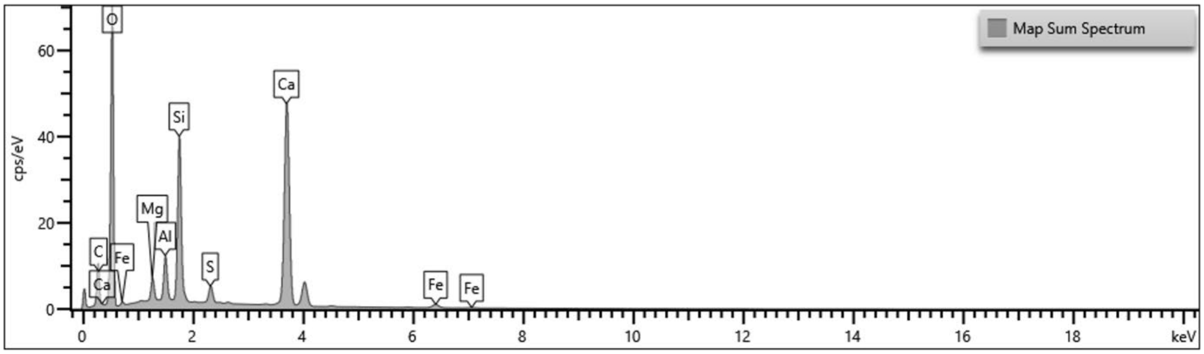

Map Sum Spectrum		
Element	Wt%	Wt% Sigma
O	51.27	0.05
Mg	1.43	0.01
Al	2.77	0.01
Si	10.43	0.02
S	1.35	0.01
Ca	30.8	0.04
Fe	1.95	0.03
Total:	100	

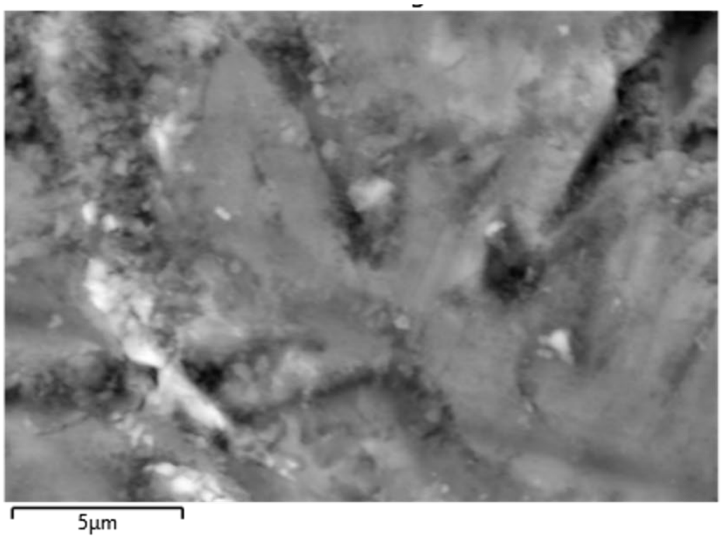

Figure S18: SEM image and EDS spectra of binder matrix in RAC-CNF-0. Site 1.

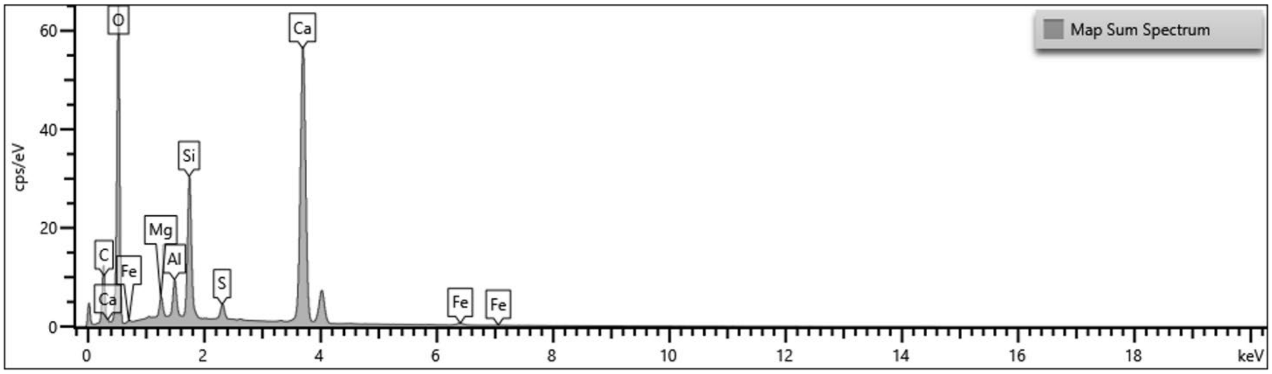

Map Sum Spectrum		
Element	Wt%	Wt% Sigma
O	51.33	0.07
Mg	1.14	0.01
Al	1.97	0.01
Si	7.66	0.02
S	1.02	0.01
Ca	35.97	0.06
Fe	0.91	0.03
Total:	100	

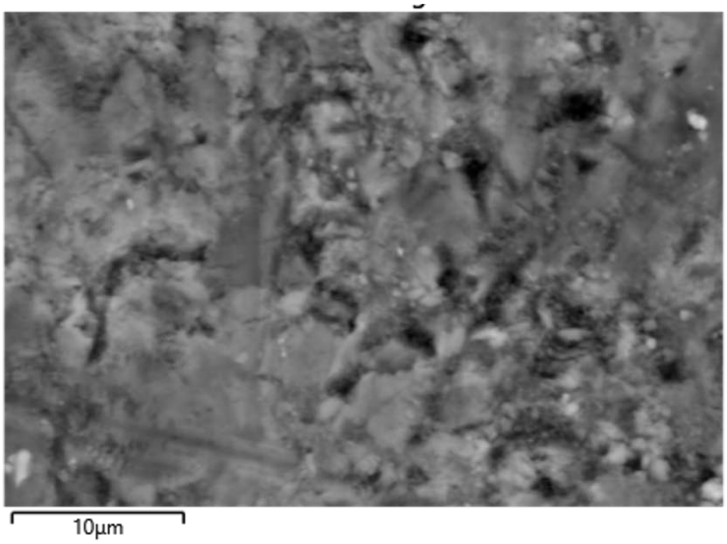

Figure S19: SEM image and EDS spectra of binder matrix in RAC-CNF-0. Site 2.

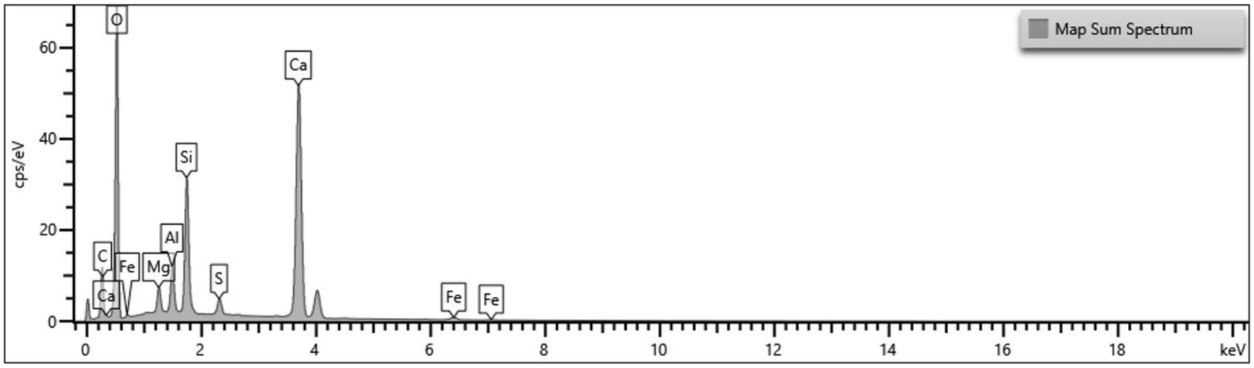

Map Sum Spectrum		
Element	Wt%	Wt% Sigma
O	51.99	0.06
Mg	1.59	0.01
Al	2.67	0.02
Si	8.06	0.02
S	1.19	0.01
Ca	33.16	0.05
Fe	1.34	0.03
Total:	100	

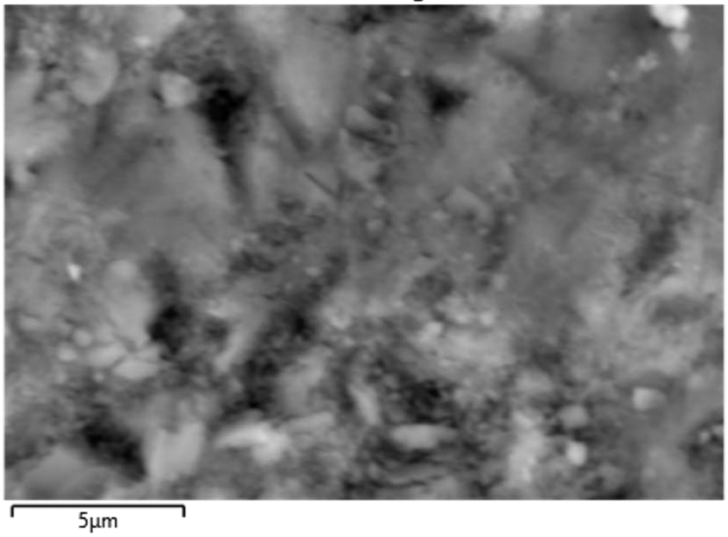

Figure S20: SEM image and EDS spectra of binder matrix in RAC-CNF-0. Site 3.

Sample RAC-CNF-0.5

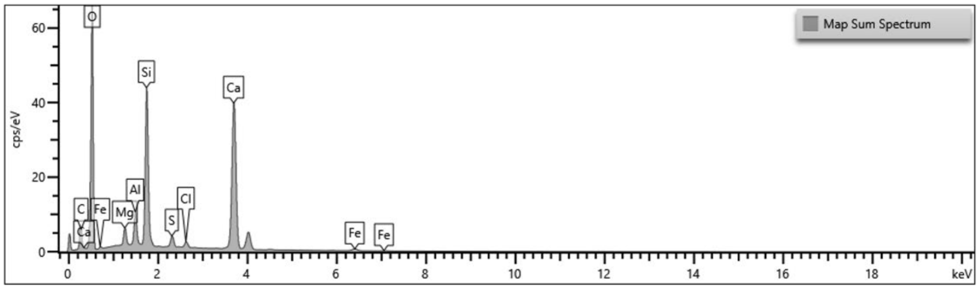

Map Sum Spectrum		
Element	Wt%	Wt% Sigma
O	51.63	0.07
Mg	1.45	0.01
Al	2.56	0.02
Si	12.62	0.03
S	1.15	0.01
Cl	0.69	0.01
Ca	28.59	0.05
Fe	1.31	0.03
Total:	100	

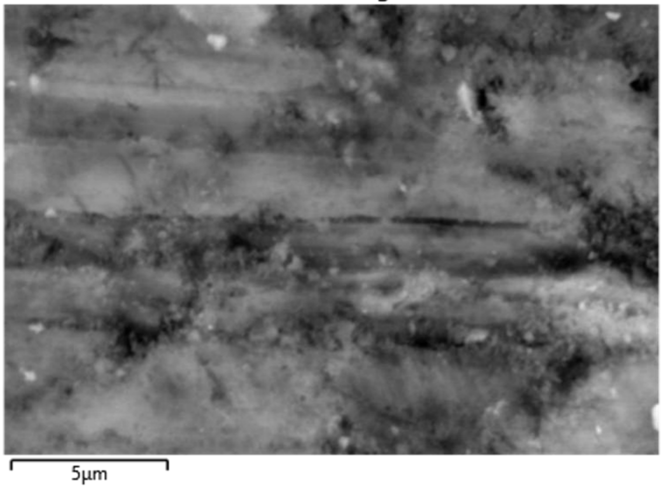

Figure S21: SEM image and EDS spectra of binder matrix in RAC-CNF-0.5. Site 1.

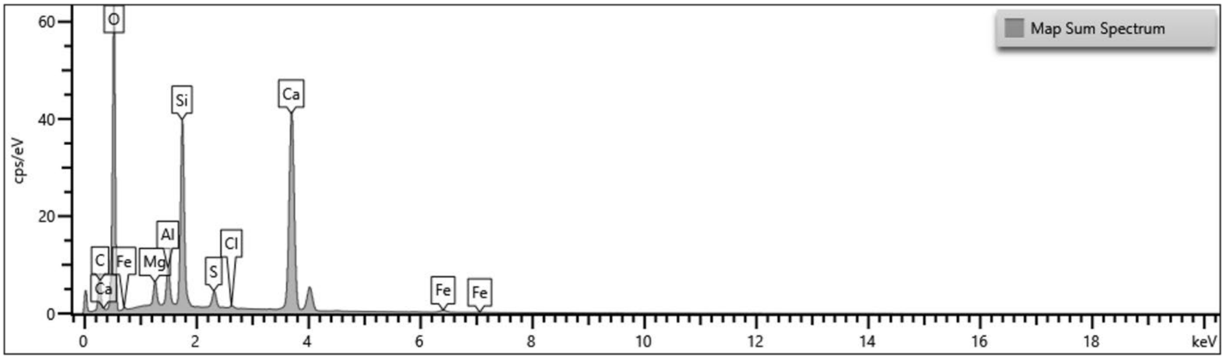

Map Sum Spectrum		
Element	Wt%	Wt% Sigma
O	51.75	0.07
Mg	1.55	0.02
Al	2.29	0.02
Si	11.6	0.03
S	1.31	0.01
Cl	0.23	0.01
Ca	30.11	0.05
Fe	1.16	0.03
Total:	100	

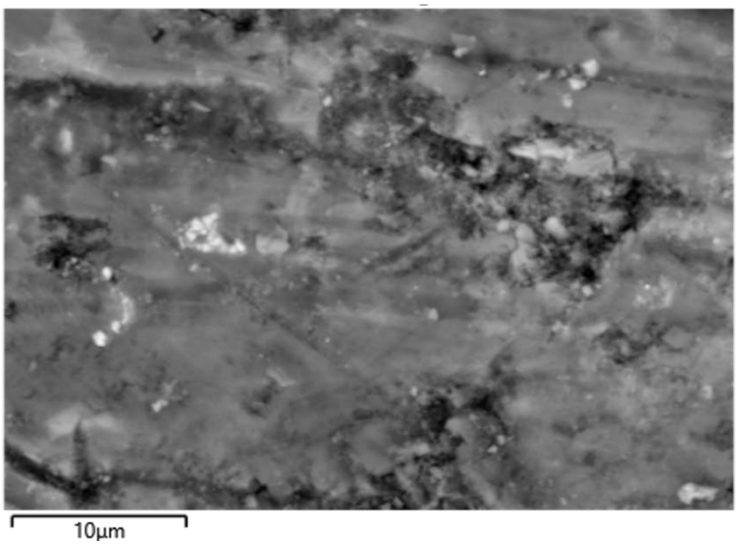

Figure S22: SEM image and EDS spectra of binder matrix in RAC-CNF-0.5. Site 2.

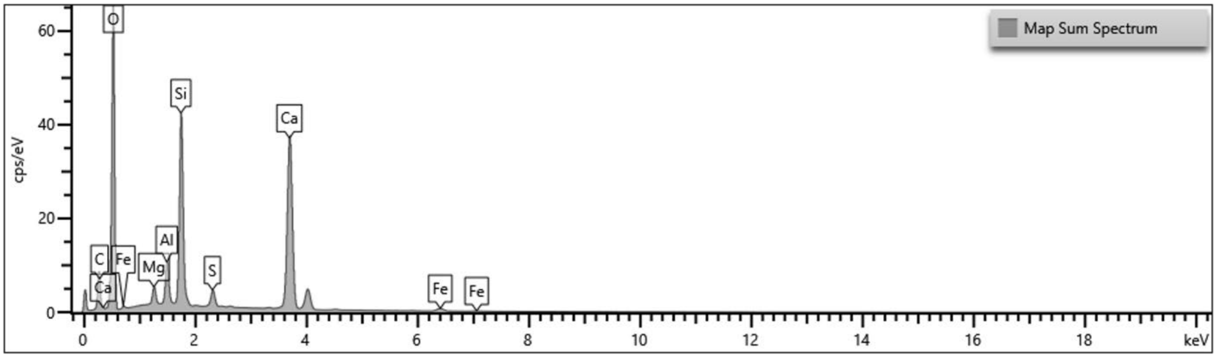

Map Sum Spectrum		
Element	Wt%	Wt% Sigma
O	52.31	0.07
Mg	1.21	0.01
Al	2.72	0.02
Si	12.77	0.03
S	1.43	0.01
Ca	27.91	0.05
Fe	1.66	0.04
Total:	100	

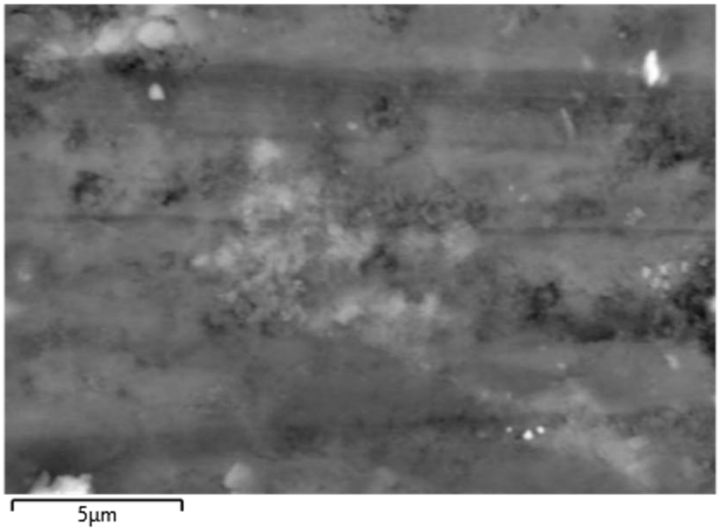

Figure S23: SEM image and EDS spectra of binder matrix in RAC-CNF-0.5. Site 3.

G. Mercury Intrusion Porosimetry

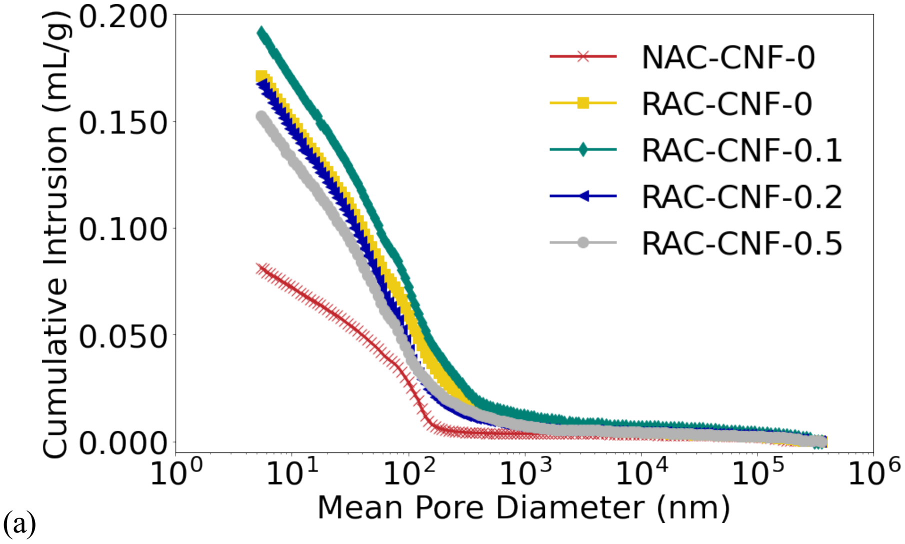

(a)

(b)

Figure S24: Cumulative pore size distributions for all mortars.

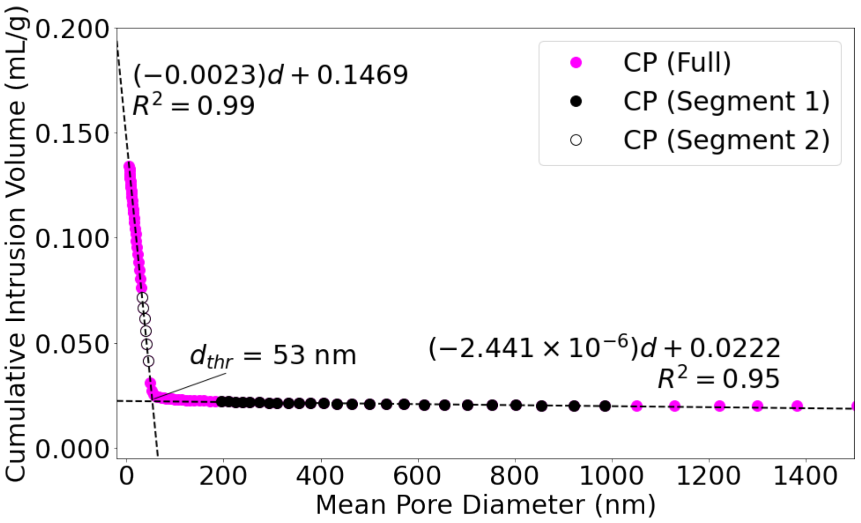

Figure S25: Determination of the threshold pore diameter for cement paste with a w/c ratio of 0.44 after 1 day of curing at room temperature and 6 days of curing in lime water; CP = cement paste; d = mean pore diameter; d_{thr} = threshold pore diameter.

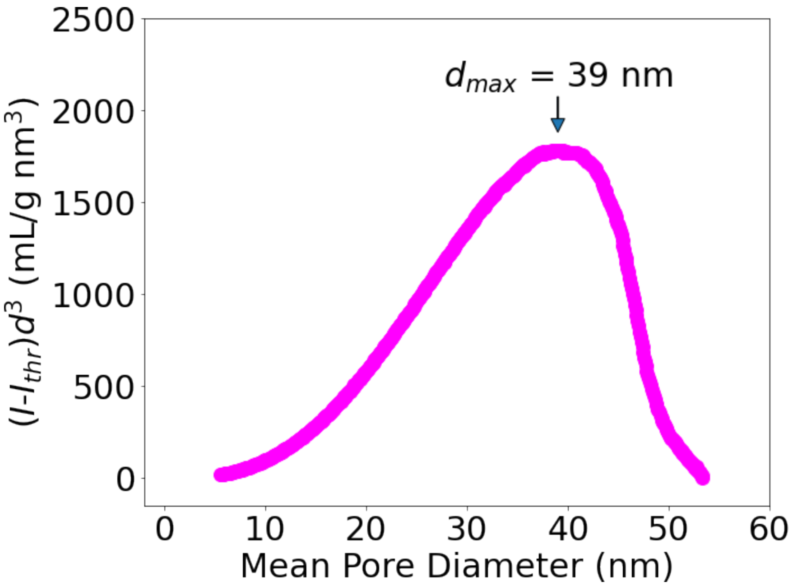

Figure S26: Determination of the pore diameter corresponding to the maximum hydraulic conductance, d_{max}, for cement paste with a w/c ratio of 0.44 after 1 day of curing at room temperature and 6 days of curing in lime water; I = specific intrusion volume at pore diameter d; I_{thr} = specific intrusion volume at the threshold pore diameter d_{thr}.

Table S4: Pore structure characteristics of cement paste with a w/c ratio of 0.44 after 1 day of curing at room temperature and 6 days of curing in lime water; d_{thr} = threshold pore diameter; k = intrinsic permeability; K = water permeability.

Porosity (%)	Skeletal Density (g/cm^3)	d_{thr} (nm)	k (m^2)	K at 20°C (m/s)
22.6	2.18	53	7.84 x 10^{-19}	7.68 x 10^{-12}

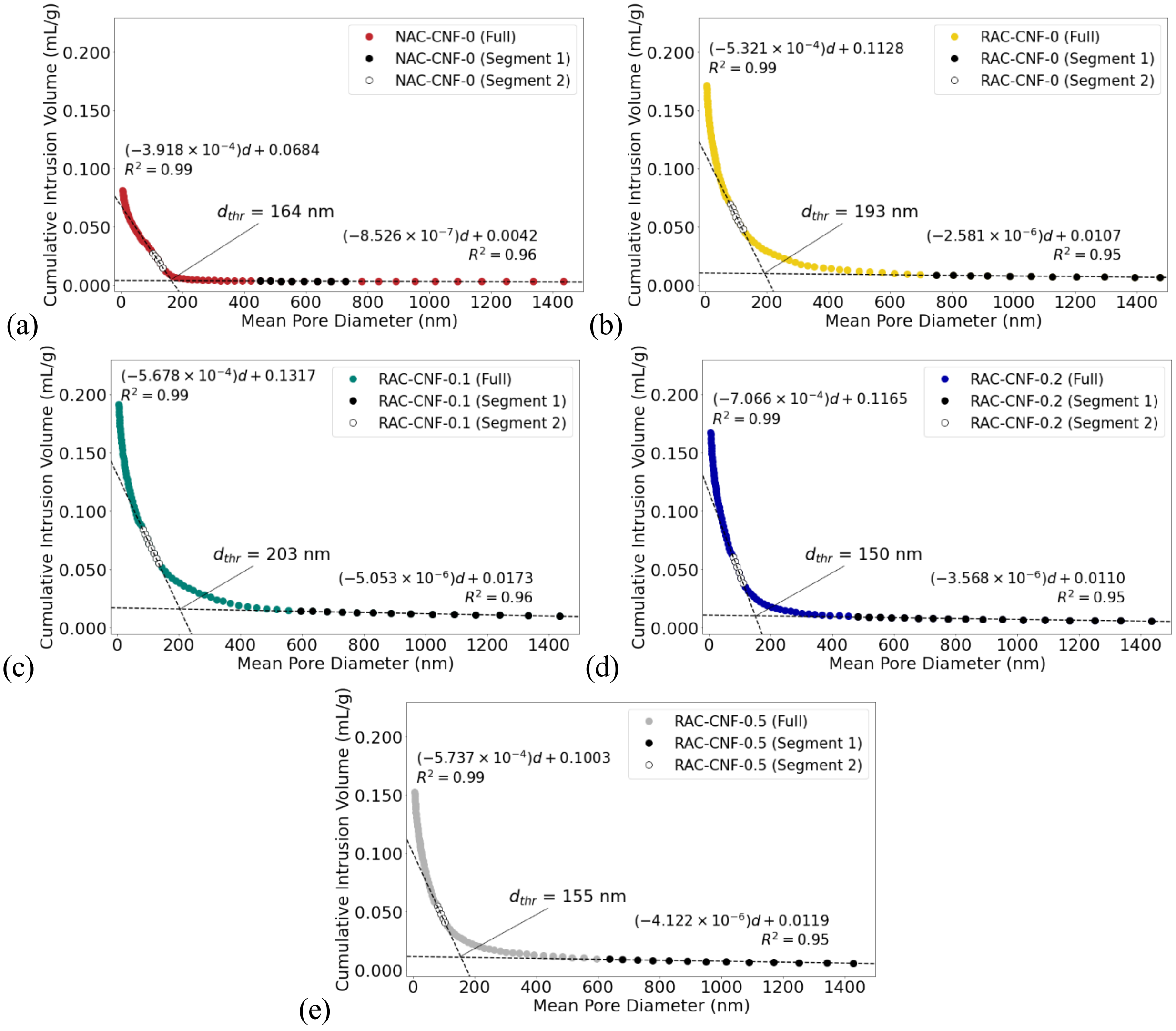

Figure S27: Determination of the threshold pore diameter, d_{thr}, for (a) NAC-CNF-0 (b) RAC-CNF-0 (c) RAC-CNF-0.1 (d) RAC-CNF-0.2 and (e) RAC-CNF-0.5; d = mean pore diameter; d_{thr} = threshold pore diameter.

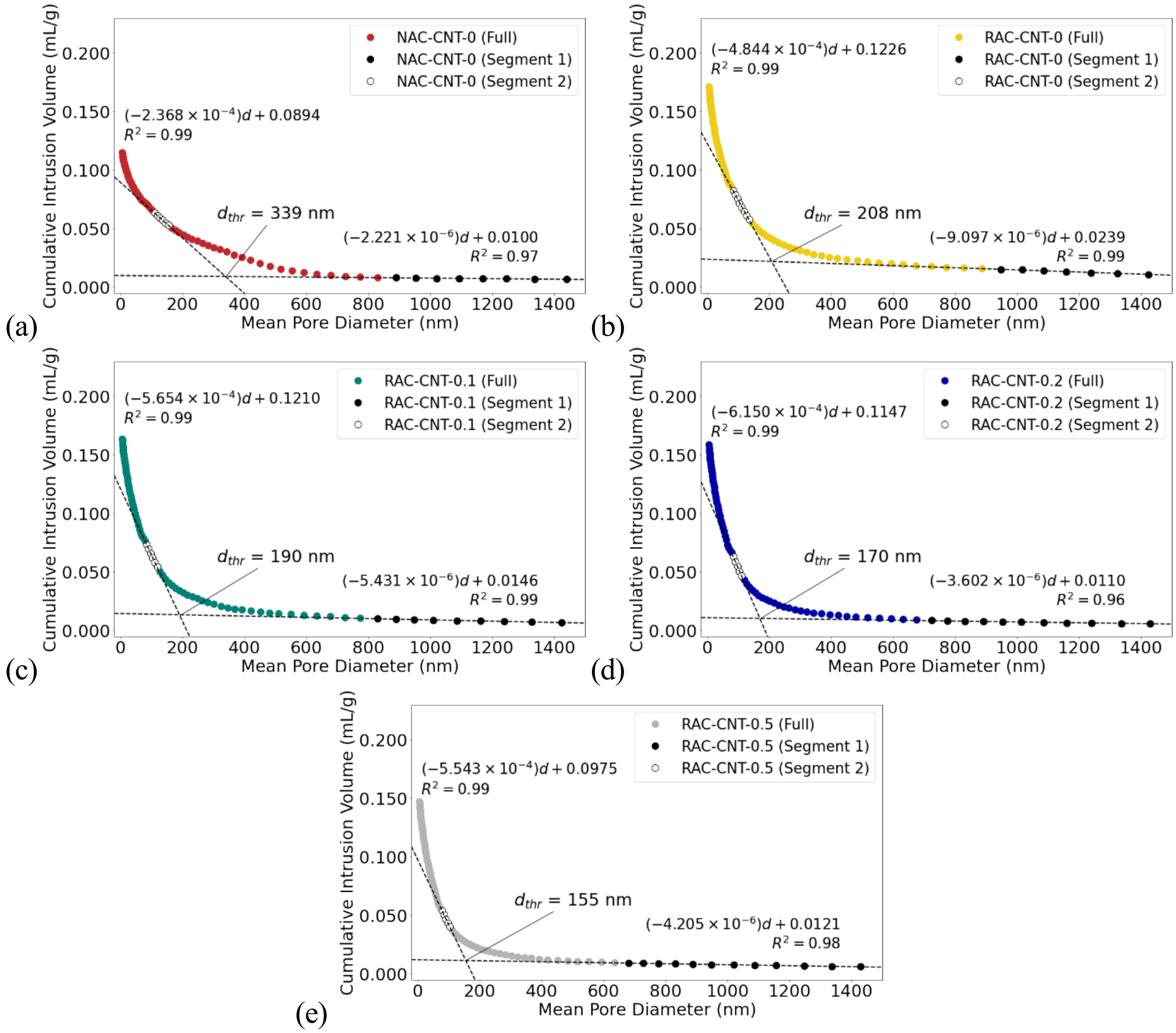

Figure S28: Determination of the threshold pore diameter, d_{thr}, for (a) NAC-CNT-0 (b) RAC-CNT-0 (c) RAC-CNT-0.1 (d) RAC-CNT-0.2 and (e) RAC-CNT-0.5; d = mean pore diameter; d_{thr} = threshold pore diameter.

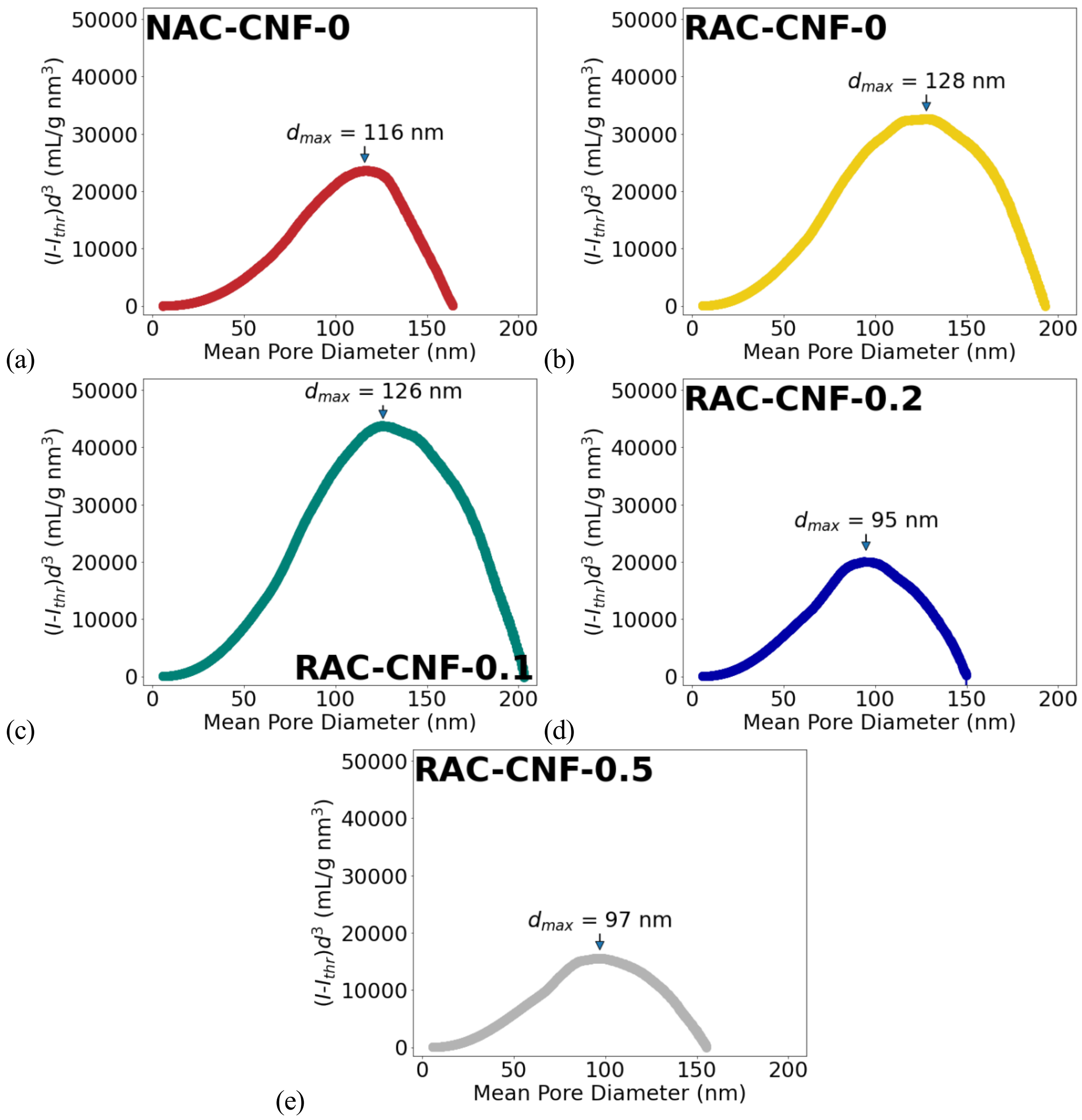

Figure S29: Determination of the pore diameter corresponding to the maximum hydraulic conductance, d_{max}, for (a) NAC-CNF-0 (b) RAC-CNF-0 (c) RAC-CNF-0.1 (d) RAC-CNF-0.2 and (e) RAC-CNF-0.5; I = specific intrusion volume at pore diameter d; I_{thr} = specific intrusion volume at the threshold pore diameter d_{thr}.
